# Recent Progress in Development of Dressings Used for Diabetic Wounds with Special Emphasis on Scaffolds

**DOI:** 10.1155/2022/1659338

**Published:** 2022-07-04

**Authors:** Ankit Awasthi, Monica Gulati, Bimlesh Kumar, Jaskiran Kaur, Sukriti Vishwas, Rubiya Khursheed, Omji Porwal, Aftab Alam, Arya KR, Leander Corrie, Rajan Kumar, Ankit Kumar, Monika Kaushik, Niraj Kumar Jha, Piyush Kumar Gupta, Dinesh Kumar Chellappan, Gaurav Gupta, Kamal Dua, Saurabh Gupta, Rohit Gundamaraju, Pasupuleti Visweswara Rao, Sachin Kumar Singh

**Affiliations:** ^1^School of Pharmaceutical Sciences, Lovely Professional University, Phagwara, Punjab 144411, India; ^2^Faculty of Health, Australian Research Centre in Complementary and Integrative Medicine, University of Technology Sydney, Ultimo, NSW 2007, Australia; ^3^Department of Pharmacognosy, Faculty of Pharmacy, Tishk International University-Erbil, Kurdistan Region, Iraq; ^4^Department of Pharmacognosy, College of Pharmacy, Prince Sattam Bin Abdulaziz University, Al Kharj, 11942 KSA, Saudi Arabia; ^5^Amity Institute of Pharmacy, Amity University Madhya Pradesh, Gwalior, Madhya Pradesh 474001, India; ^6^Department of Biotechnology, School of Engineering & Technology (SET), Sharda University, Plot No. 32-34 Knowledge Park III, Greater Noida, Uttar Pradesh 201310, India; ^7^Department of Life Sciences, School of Basic Sciences and Research, Sharda University, Plot No. 32-34, Knowledge Park III, Greater Noida, 201310 Uttar Pradesh, India; ^8^Department of Biotechnology, Graphic Era Deemed to be University, Dehradun, 248002 Uttarakhand, India; ^9^Department of Life Sciences, School of Pharmacy, International Medical University, Bukit Jalil, 57000 Kuala Lumpur, Malaysia; ^10^School of Pharmacy, Suresh Gyan Vihar University, Mahal Road, Jagatpura, Jaipur, India; ^11^Department of Pharmacology, Saveetha Dental College, Saveetha Institute of Medical and Technical Sciences, Saveetha University, Chennai, India; ^12^Uttaranchal Institute of Pharmaceutical Sciences, Uttaranchal University, Dehradun, India; ^13^Discipline of Pharmacy, Graduate School of Health, University of Technology Sydney, NSW 2007, Australia; ^14^Chitkara College of Pharmacy, Chitkara University, Punjab, India; ^15^ER Stress and Mucosal Immunology Lab, School of Health Sciences, University of Tasmania, Launceston, Tasmania, Australia 7248; ^16^Department of Biomedical Sciences and Therapeutics, Faculty of Medicine and Health Sciences, Universiti Malaysia Sabah, Kota Kinabalu, 88400 Sabah, Malaysia; ^17^Centre for International Relations and Research Collaborations, Reva University, Rukmini Knowledge Park, Rukmini Knowledge Park, Kattigenahili, Yelahanka, Bangalore, 560064, , Karnataka, India

## Abstract

Diabetic wound (DW) is a secondary application of uncontrolled diabetes and affects about 42.2% of diabetics. If the disease is left untreated/uncontrolled, then it may further lead to amputation of organs. In recent years, huge research has been done in the area of wound dressing to have a better maintenance of DW. These include gauze, films, foams or, hydrocolloid-based dressings as well as polysaccharide- and polymer-based dressings. In recent years, scaffolds have played major role as biomaterial for wound dressing due to its tissue regeneration properties as well as fluid absorption capacity. These are three-dimensional polymeric structures formed from polymers that help in tissue rejuvenation. These offer a large surface area to volume ratio to allow cell adhesion and exudate absorbing capacity and antibacterial properties. They also offer a better retention as well as sustained release of drugs that are directly impregnated to the scaffolds or the ones that are loaded in nanocarriers that are impregnated onto scaffolds. The present review comprehensively describes the pathogenesis of DW, various dressings that are used so far for DW, the limitation of currently used wound dressings, role of scaffolds in topical delivery of drugs, materials used for scaffold fabrication, and application of various polymer-based scaffolds for treating DW.

## 1. Introduction

Diabetic wound (DW) is one of the complications of diabetes that affects the quality of life of patients. Its cases are continuously increasing at a rapid rate. The lack of glycaemic control, hypoxia, vasculopathy, immunopathy, and damage to the cells due to the release of reactive oxygen species (ROS) contribute towards DW [1, 2]. It affects 42.2 % of diabetics and imposes an economic burden on the health sector that bears a cost of 3 billion dollars for the treatment every year [3]. Moreover, in DW, sequences of wound healing are compromised especially the inflammatory phase. The delay in wound healing is due to the inability of platelets, macrophage growth factors (GFs), cytokines, and chemokines to act normally upon the cellular receptors. This leads to abruption in signalling cascades, which in turn results in impairment of angiogenesis, collagen synthesis, collagen proliferation, differentiation, and migration and impedes the wound healing cycle. This can further lead to amputation or death of the patient. The global prevalence rate of amputation due to DW is 7-20% [4, 5].

It has been observed that the wound of patients suffering from DW looks like an ulcer, especially on the feet and lower extremities. The presence of exudate and bacterial load at the wound site makes the management of DW more complicated and further leads to infection. To combat with this complication an appropriate treatment strategy is required. Till date, various wound dressings have been explored which, include gauze, foams, films, hydrocolloids, iodine dressings, silver dressings, polysaccharide dressings, natural polymer, and synthetic polymer-based dressing. However, these treatment strategies are unable to provide adequate patient compliance and are associated with various limitations. In recent years, scaffolds have gained attention as new dressing and provide new dimension in the field of tissue rejuvenation.

Scaffolds are three-dimensional polymeric structures formed from polymers that help in tissue rejuvenation. Moreover, scaffolds are versatile in use, offer controlled size, tunable physicochemical properties, and offer a large surface area to volume ratio to allow cell adhesion and exudate absorbing capacity, antibacterial properties, and encapsulation of drug for the desired period [6]. Their ability to encapsulate drugs for desired period helps in achieving their controlled release. This release controlling property is neither provided by any of the dressings mentioned above nor by existing novel drug delivery systems (e.g., liposomes, nanostructured lipid carriers (NLCs), nanoparticles (NPs), and dendrimers) used for topical application. Looking at their enormous benefits, an attempt has been made to explore and summarize various scaffolds to treat DW [7].

The present review provides a comprehensive overview of global prevalence of diabetic wound, their etiopathogenesis, various conventional and smart dressings, and advantages of scaffolds over other delivery system, role of scaffolds as a topical drug delivery, their application in treating diabetic wound, modifications in the scaffolds for the management of DW and market as well as clinical status of scaffolds.

## 2. Pathogenesis of DW

In patients having long history of uncontrolled DM, the high blood glucose level results in vasculopathy, immunopathy, and neuropathy. These further caused by alteration in the normal wound healing pathways. The pathways which are affected in DW are aldose reductase sorbitol dehydrogenase hexosamine pathway and nitric oxide synthase pathway. The alteration in aldose reductase sorbitol dehydrogenase pathway results in increase in sorbitol and fructose. This further leads to decrease in the level of myoinositol that further results in generation of neuropathy. Due to this, cell chemotaxis, GF production, and cell proliferation decrease, which impedes the wound healing process. Higher blood sugar also activates hexosamine pathway, which leads to decrease in levels of glucose-6-phosphatase dehydrogenase and nicotinamide adenine dinucleotide phosphate (NADPH). These cause decrease in levels of myoinositol and nitric oxide and lead to generation of ROS. Thus, the wound healing process gets delayed due to oxidative stress. The inhibition of nitric oxide synthase enzymes results in decrease in the level of nitric oxide that leads to oxidative stress. The overall pathogenesis causes decrease in angiogenesis [8], platelet function, and increase in inflammation as well as vasoconstriction at the site of wound and impedes the normal wound healing process [9–11].

Besides these, during hyperglycemia, the levels of miRNA-146a and miR-132 get decreased, whereas the level of miR-155 gets overexpressed. The suppression of miRNA-146a increases the proinflammatory mediators such as interleukin-1 receptor- (IL-1R-) associated kinase (IRAK1) and tumour necrosis factor (TNF) receptor-associated factor 6 (TRAF6). These extend the inflammatory phase and impede the wound healing process [12]. The reduction in the level of miR-132 expression also delays the wound healing process by increasing the levels of nuclear factor kappa-light-chain-enhancer of activated B cells (NF-*κΒ*), nucleotide-binding oligomerization domain- (NOD-) like receptor, Toll-like receptors (TLR), and TNF signalling pathway. The increase in levels of these proteins results in a prolonged inflammatory phase by releasing various inflammatory mediators from macrophages and monocytes and delays the wound healing process. Furthermore, miR-132 also targets the heparin-binding EGF-like GF (Hb-EGF) and facilitates the transition from the inflammatory phase to the proliferative phase [13]. On the other side, the overexpression of miR-155 leads to an increase in myeloperoxidase peroxidase- (MPO-) positive cells and decrease angiogenic markers suggestive of ECM build-ups such as TGF-*β*1, collagen 1, and alpha-smooth muscle actin (*α*-SMA) [13]. Overall, these cause the generation of DW. In another study, it was found that an increase in levels of miR-191 and miR-200b was positively associated with higher levels of inflammation-associated markers such as C-reactive protein (CRP) which results in reduced tube formation capacity, migration, and zona occludens-1 expression in human dermal endothelial cell and impede wound healing process [14]. The increase in levels of miR-26a also influences the DW healing process by targeting small mothers against decapentaplegic-1 (SMAD-1) and impairs wound edge angiogenesis and granulation tissue thickness. The other nucleic acids implicated in DW healing are miR-15b, miR-200, and miR-205–5p. The increase in levels of these nucleic acids results in the deactivation of vaso-endothelial growth factor (VEGF) pathways and impairs the wound healing process [15].

Activation of VEGF-AKT/enNOS (protein kinase B/nitric oxide synthase) pathway involves in the angiogenesis and helps in wound healing. But at the same time during injury, the level of miR-615-5p gets increased which acts as negative regulator of the angiogenesis and wound healing by inhibiting the activation of VEGF-AKT/enNOS with the help of other genes (IGF2 and RASSF2). Hence, by neutralizing the miR-615-5p, the process of angiogenesis and wound healing can be promoted [8].

**Figure 1:**
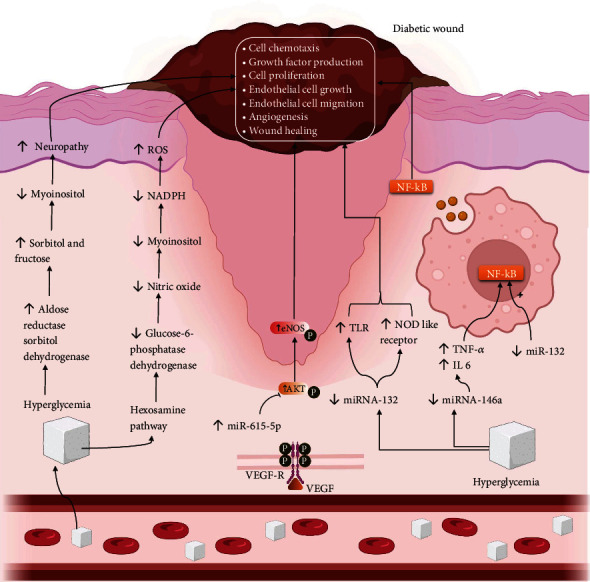
The pictorial representation of pathogenesis of diabetic wound due to hyperglycaemia leading to oxidative stress, neuropathy, immunopathy, and vasculopathy.

The hyperglycemia further results in idiopathic complications, viz., neuropathy, immunopathy, and vasculopathy. Neuropathy is a common complication of different medical conditions. It affects autonomic nerves, motor nerves, and sensory nerves. In motor neuropathy, weakness and wasting of intrinsic foot muscles take place that finally leads to ulceration. In sensory neuropathy, loss of pain occurs, leading to unnoticed trauma, which in turn gives rise to ulcer formation. In autonomic neuropathy, sweating is decreased due to which skin becomes dry and brittle. This leads to secondary infection and finally causes ulceration. Vasculopathy is a general term used to describe any disease affecting blood vessels. It is of two types, microangiopathy and macroangiopathy. Macroangiopathy occurs due to the deposition of blood clots and fats in the blood vessels. Obstruction in the blood flow leads to tissue necrosis and finally, ulceration takes place. In the case of microangiopathy, more glycoproteins are formed on the surface of the basement membrane due to which vessel walls grow abnormally thicker and weaker leading to disruption of vessels. It causes the leakage of blood and proteins and also slows down the flow of blood to different parts of the body. This is another cause of ulcer formation. In immunopathy, polymorpho-nuclear leukocyte migration, phagocytosis, chemotaxis, and intracellular killing rate get decreased. Decreased chemotaxis of GFs and cytokines, coupled with an excess of metalloproteinases, impedes normal wound healing by creating a prolonged inflammatory state [16–19]. The pictorial representation of pathogenesis of DW is shown in Figure 1.

## 3. Current Wound Dressings for DW

The various wound dressings which have been explored to treat DW include gauze, foams, films, hydrocolloids, iodine dressings, silver dressings, polysaccharide dressings, and natural polymer- and synthetic polymer-based dressing. The alternatives of these dressings are smart dressings which are discussed below.

Gauze-based dressings require continuous replacement, and upon removal, particulates remain at the wound bed that reinjure the wounds. Furthermore, the mechanical removal of gauze-based dressings from the wound bed eliminates healthy as well as unhealthy tissues; thus, it is not considered as the safest dressing [20, 21]. Moreover, foam-based dressings and adherent contact layer-based dressings need secondary dressing because they can dry after a shorter period and are not appropriate for dry wounds. Additionally, these dressings are less stable, having an unpleasant odour as well as cause pain during their removal. Furthermore, these impart easy invasion of bacteria and infection at the wound site [22–24]. Film-based dressings overcome the limitations of foam-based dressings such as odour, stability, and infections. However, these are not appropriate for wounds with high exudate and neuropathic ulcers. Similarly, hydrocolloid-based dressings are ineffective against high exudate, need continuous changing, offer pain, macerate the wound, and not safe for dry wounds [25].

Natural polymer-based dressings include alginate, chitosan, collagen, and cellulose. Alginate-based dressings are much better than hydrocolloid dressing due to pain controlling ability, ease to use, and exudate absorbing capacity [26]. The limitation associated with these types of dressings are associated high cost and maceration of wound [27]. Iodine-based dressings are toxic for keratinocytes and fibroblasts. Additionally, they can impart discolouration of the wound. Silver-based dressings are widely accepted in wound healing due to their antimicrobial actions. Moreover, in one of the studies, it has been found that silver-based dressing provided 100% granulation tissue formation at the wound site as compared to iodine-based dressing. This indicated the advantage of silver dressing over the iodine-based dressing [28]. The main disadvantage associated with silver-based dressings is staining of silver to the wound. Hence, it is not widely used in DW treatment. Natural polymer-based dressings are much effective than the aforementioned dressings. Firstly, cellulose dressings are cost-effective, release GFs to provide a proliferation of fibroblasts at the wound site, and provide a moist environment to the wounds. The limitation of this type of dressing is lack of antimicrobial activity and overabsorbed by the excess exudate present at the wound bed [29].

Chitosan dressings are much effective than cellulose dressings due to their antimicrobial and hemostatic properties. The limitation associated with these dressings is extensive swelling in water and the inability to dissolve in the organic solvents due to their rigid crystalline structure [30]. Collagen and gelatin combination-based dressings promote angiogenesis and granulation at the site of injury. Moreover, they can exhibit anti-inflammatory as well as antimicrobial properties. Hyaluronic acid-based dressings provide lubrication and water absorptive properties. Furthermore, they can help in collagen deposition, epithelial migration, and angiogenesis at the wound site. In one of the studies, it has been found that only low molecular weight hyaluronic acid helps in tissue rejuvenation, whereas high molecular weight hyaluronic acid inhibits angiogenesis as well as restricts the supply of nutrients to the tissues which impede the wound healing process [31].

The synthetic dressings used for wound healing include poly (lactide-co-glycolide), polyurethanes, and polyethene glycols. Synthetic polymer dressings are much considerable than natural polymer dressing due to their consistency, reproducible physical as well as chemical composition. Poly (lactide-co-glycolide)-based dressings are approved by the Food and Drug Administration (FDA) for suture as well as drug delivery applications. The variation in lactide to glycolide results in the release of GFs that stimulates wound healing. Furthermore, they are cytocompatible and promote fibroblasts proliferation, spreading, and adhesion. The limitation associated with this dressing is the need for external antimicrobial agents, and its properties do not match with extracellular matrix and collagen [31]. Polyurethane dressings consist of a semipermeable membrane that restricts the entry of pathogens towards the wound site as well as provides a moist environment and drainage properties to the wound that helps in reducing swelling. The drawback of this type of dressing is requirement of additional composite dressings in order to provide contact layer and waterproof properties [29]. In contrast, poly (ethylene glycol) dressings are flexible, biocompatible, and hydrophilic in nature. Additionally, they provide better grip in the contact layer and have higher affinity for GFs. However, their major limitation includes damage of granulation tissues due to the adhesive used in the dressings. Polycaprolactone-based dressings are having structure similar to the extracellular matrix architecture. These absorb the exudate and provide controlled release and slow degradation. The drawback of this type of dressings is absence of antimicrobial properties [29–32].

These types of traditional dressings can protect the wounds from the external environment but do not respond to the wound healing process well. The advanced forms of these dressings are smart wound dressings that can help in tissue rejuvenation by showing interaction with the wound, where they can sense and react with the wound environment and promote wound healing. The presence of smart materials such as stimuli-responsive materials and self-healing materials and in-built sensors makes them as an ideal tool in the management of wounds. The various smart dressings used till now to manage wounds include self-healing wound dressing, stimuli-responsive wound dressing, biomechanical wound dressing, monitoring wound dressing, and self-removable wound dressing.

Self-healing wound dressing is generally used for motional wounds where the chances of mechanical stress are very much which leads to split of dressings from the wound site. Researchers have developed self-healing wound dressings which have the ability to tolerate the mechanical stress and intact the wound dressings at the wound site for a prolonged period of time. For instance, in one of the study, researchers developed a self-healing hydrogel of curcumin-quaternized chitosan crosslinked benzaldehyde-terminated Pluronic F127. This type of wound dressing was suitable for motional injuries due to their stretchability and adhesive nature. Moreover, these dressings were not split from the elbow site at 120°C. In addition, these dressings were biodegradable and did not require changing during the service period. In another study, Li et al. prepared a self-conductive hydrogel dressing of cationic guar slime (GS) and poly (3, 4-ethylenedioxythiophene) : poly (styrene sulfonate) (PEDOT : PSS) for motional wounds. This type of wound dressing was applied to the neck of the rats where lots of tension, twist, and stress occur within a minute. The results showed that this type of wound dressing bear stretching upto 200% and promotes deposition of collagen and granulation of tissue at the site of injury. Till date, many self-healing dressings are developed but are not available for the management of motional wounds. In addition to the self-healing ability and good adhesive capacity of self-dressings, researchers should emphasize on mechanical properties of dressings to overcome stress associated with motional wounds.

The removal of dressings from the wound site offers extreme pain and reinjury at the wound upon its removal. To overcome these challenges, self-removal dressings have been developed. Numerous strategies have been used by the researchers to develop these dressings. The first one is the use of thermosensitive polymer that becomes liquid at a particular temperature and offers ease of removal of dressing. For instance, Wagner and coworker used polyisocyanopeptide (PIC) as thermosensitive polymer for the development of self-removal dressings. The polymer used in this dressing becomes liquid below 16°C and underwent gelation into hydrogel at room temperature. Despite being effective, these dressings require a particular temperature for easy removal of dressings from wound area. Therefore, this is considered a major drawback of thermosensitive polymer-based self-dressings and also cannot be used under cold conditions. To address these challenges, self-removal dressing was prepared by using PEG-thiols and oxidized dextran which results in the formation of a hydrogel. To break bonds present in between, this hydrogel glutathione or cysteine can be added which results in easy removal of dressing from the wound site. The other approach used by the researchers for designing self-removal dressings is light-triggered dissolution. In this approach, Wu et al. prepared a UV-triggered on-demand dissolution self-healing wound dressing by using glycol chitosan (GC) and polyethylene glycol (PEG)-4-(3-(1-(Nhydroxysuccinimidyl carbonic ester)ethyl)-4-nitrophenoxy) butanoate (PNN). The crosslinking between GC and PNN was due to reaction between the amino group present in the glycol chitosan and the N-hydroxysuccinimidyl carbonate in PNN. PNN is photocleavable because of its ortho-Nitrobenzyl (o-NB) groups, which are sensitive to UV light. Since light is a noninvasive stimulus, this wound dressing can be removed from the wound site in a noninvasive manner [33].

Wound healing is a normal physiological and biological process that involves a series of physical as well as chemical changes at the site of injury. During injury, there are numerous factors such as temperature, pH of the wound site, glucose level, and oxygen level which play a key role in wound healing. Looking at these factors, researchers developed stimuli-responsive wound dressings that can respond to these changes and can effectively manage the wound. A summary of the stimuli-responsive wound dressings, their advantages, and limitations can be found in Table 1.

## 4. Role of Scaffolds in Topical Delivery of Drug

Topical route is the most preferred route to target drugs at the wound site. However, this route also faces some challenges in delivering drugs due to the enzymes present in epidermal layer of skin and other layers of skin that impediment permeation of drugs across them. This becomes more cumbersome if they are unable to cross these barriers. To overcome these challenges, various novel drug delivery systems have been formulated such as NLCs, solid lipid nanocarriers (SLNs), NPs, micelles, and liNot Availableposomes etc. They have been reported to enhance the permeability of drug across the skin via transcellular route, paracellular route, and endocytosis owing to their nanometer size and elasticity. Thus, they provide target specificity and release the drug at the wound site. In transcellular route, drug directly passes from the lipid layers, whereas in paracellular route, drug reaches at the target site by passing through the tight junctions present in the skin that is usually not observed with drugs present in conventional formulations. In endocytosis, drugs reach the wound site by entering through the pores present on skin [35, 36]. The aforementioned nanocarriers have been successfully utilized in treating DW; however, they retent at the wound site for small time. The challenges related to poor retention of drugs by the aforementioned nanocarriers can be overcome by loading/implementing them into dressings/scaffolds [36]. This further improves their drug delivery efficacy. Scaffolds are biodegradable in nature and upon their application at the wound site, they start degrading and releasing the drug in a time-dependent manner from the nanocarriers. Thus, they provide high retainability of drug at the wound site and target the pathways that are responsible for impeding wound healing process [37]. In addition, they help in tissue regeneration that helps in faster wound healing. In recent years, there are many studies that have been loaded into the dressings/scaffolds for effective treatment of DW. Some of the latest studies on nanocarriers implemented in dressings/scaffolds are mentioned in Table 2.

**Figure 2:**
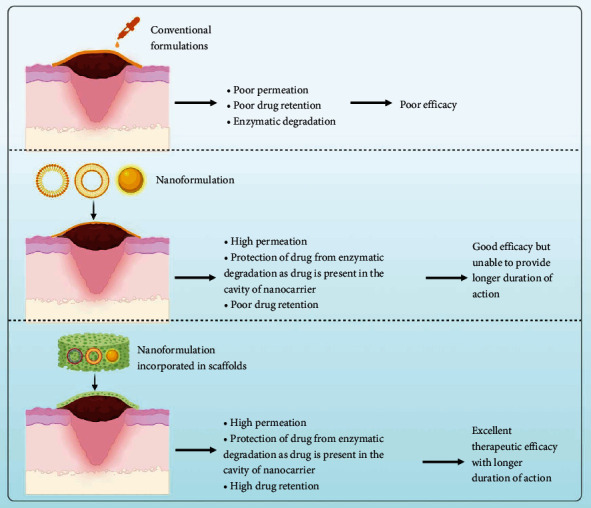
The limitations of conventional therapy in wound healing and advantages of nanoformulation-loaded scaffolds over conventional formulation as well as nanoformulation without scaffolds.

The advantages of nanoformulation-loaded scaffolds over conventional formulation and nanocarrier nonloaded in scaffolds are shown in Figure 2.

## 5. Materials Used in Scaffold Fabrication

The materials used in scaffold fabrication are derived from natural sources such as plants and animals as well as manufactured synthetically. These biomaterials must be biodegradable, biocompatible, and free from antigenicity and inflammatory response after implantation. Various natural/synthetic polymers (Table 3) that are used to fabricate scaffolds are discussed in the following sections.

### 5.1. Natural Polymers

The source of natural polymers are plants and animals. They are more biocompatible than synthetic counterparts due to the presence of tripeptide arginine-glycine-aspartate (RGD) sequences. These sequences help in cell attachment and acts as a receptor for cell adhesion molecules. Natural polymers are also extracted from tissues and utilized as a seminatural matrices by separating cellular materials from them, e.g., decellularized umbilical cords. The extraction of natural polymer is a tedious process and having higher cost as compared to synthetic polymers [69]. Various natural polymers used in scaffold fabrication are discussed in the subsequent sections.

#### 5.1.1. Polysaccharides

Polysaccharides are major class of biomolecules with long chain carbohydrate subunits. They are formed by combination of several monosaccharides. They are mostly used as starting materials for the preparation of tissue scaffolds. The unique feature of polysaccharide-based scaffolds is to form thermoreversible elastic hydrogel and having low modulus that helps in soft tissue rejuvenation such as skin. Some of the polysaccharides used in tailoring scaffolds include alginate, chitosan, cellulose, iota-carrageenan, konjac gum, xanthan gum, and kappa carrageenan. Besides these, other polymers used to fabricate scaffolds are pullulan, starch, dextran, and cellulose. Among these polysaccharides [70], chitosan [71], alginate [72, 73], konjac gum [74, 75], cellulose [76–78], *Ganoderma lucidum* [74, 75], and *β*-glucan [79–81] based scaffolds have been utilized for DW. These have been discussed in the subsequent sections.

The biological sources of alginates are brown seaweeds. These are anionic in nature and consist of copolymers of D-mannuronic acid (M monomer) and L-guluronic acid (G monomer). Alginates are gaining remarkable attention in tissue engineering due to their less toxicity, biocompatibility, relatively less cost, and ease of gelation. Furthermore, alginate-based biomaterials are not only used in the drug delivery system but also used as cell carrier. Additionally, alginates are also utilized as a good therapeutic agent in wound healing due to their anti-inflammatory, analgesic action, and structural similarity with extracellular matrix (ECM) [82].

Chitosan is a cationic linear polysaccharide with high molecular weight. Commercially, it is manufactured by deacytelyation of polyacetylglucosamine found in walls of fungi, shrimps, and other crustacean shells. It mainly consists of D-glucosamine and N-acetyl-D-glucosamine. Chitosan is widely accepted as biomaterial for fabricating scaffolds due to its biodegradability, antibacterial effect, nontoxicity, and biocompatibility that arise due to the presence of primary amines in them [83]. Furthermore, it helps in stimulation of haemostasis and accelerates wound healing [84, 85]. In addition, chitosan has also structural resemblance with glycosaminoglycans that helps in formation of ECM. ECM helps in the migration of keratinocytes and promotes collagen deposition at the wound site that helps in wound healing. Moreover, the unique biological properties of a chitosan-based hydrogel enable it to serve as both a wound dressing and as a drug delivery system (DDS) to deliver antibacterial agents, GFs and stem cells which could further accelerate wound healing [86].

Konjac gum is a reserve polysaccharide present in the cell-wall of *Amorphophallus konjac*. It is a linear chain carbohydrate polymer consisting of 1,4-*β*-linked D-mannosyl and D-glucosyl residues. It exhibits intrinsic biological activities and excellent thickening and water binding properties [87]. It is used in the fabrication of scaffolds due to its biocompatibility, nonimmunogenicity, and nontoxicity. In addition, konjac gum is used in tissue engineering because of its structural similarity with ECM and provides moist environment at the site of injury. Moreover, konjac gum also helps in fibroblasts proliferation and attachment, migration of keratinocyte, and collagen expression at the site of injury and accelerates the wound healing process [88].

Cellulose is a natural complex carbohydrate found in plants and algae. It mainly consists of D-glucose units linked by *β* (1→4) linkage. It is gaining remarkable attention as a biomaterial because of its flexibility, functionality, biodegradibility, and biocompatibility. Moreover, it exhibits tunable physico-chemical features such as porosity [89]. It is used in tissue engineering due to its thermo-gelling ability, high-water retention capacity, anti-inflammatory action, high surface area to volume ratio, angiogenesis, collagen deposition, and epithelization that accelerates the wound healing process [90].

*Ganoderma lucidum* is a medicinal fungus belonging to family Ganodermataceae. It has many therapeutic benefits due to which it is known as “mushroom of immortality.” It is a pyranoid glucan with beta-glycosidic bond. It is used as a tissue rejuvenator due to its antimicrobial, immunomodulator, and antioxidant effects. Moreover, it is used in DW healing by extracting various phytoconstituents from it such as total polysaccharides (25.1%), ganoderic acid A (0.45%), and adenosine (0.069%). These phytoconstituents play a vital role in DW healing by decreasing oxidative stress, increase collagen deposition, immunomodulation, and reepithelization at the site of injury [91, 92].

Various case studies where polysaccharides have been utilized to fabricate scaffolds are discussed below. These scaffolds have been utilized to deliver drugs, drug loaded in nanocarriers as such as NPs, hydrogel, and NLCs.

Activity of chitosan-collagen-based scaffolds loaded with thymosin *β*-4 (CCSS-eT*β*4 scaffolds) was checked against DW in streptozotocin- (STZ-) induced diabetic rats and were compared with CCSS loaded with infiltrate T*β*4 scaffolds (CCSS-iT*β*4 scaffolds). The prepared scaffolds were also tested for *in vitro* drug release study in order to evaluate the sustained release profile. The results of vitro drug release study revealed that CCSS-eT*β*4 scaffolds exhibited initial rapid release of T*β*4 (67.7%) within 4 days followed by a linear and steady release of T*β*4 (92.6%) in 12 days indicating better accumulation release of T*β*4, whereas about 45% release of T*β*4 in first 4 days and 85.3% at the end of the 12^th^ day. The authors reported controlled release of T*β*4 from CCSS-eT*β*4 scaffolds was achieved due to drug diffusion and interaction between the peptide and scaffolds. In addition, topical application of prepared CCSS-eT*β*4 scaffolds showed 1.2-folds and 10-folds decrease in wound area and IL-6 levels as compared to CCSS-eT*β*4 alone-treated group [93].

Intini et al. studied the effect of 3D chitosan-based scaffolds against DW in STZ-induced female Wistar diabetic rats. The study was conducted for 14 days and results of wound closure were noted. Further, the results were compared with diabetic control and commercial dressings (carboxymethyl cellulose-based dressings). It was found that 3D chitosan-based scaffolds showed 1.1-folds and 2.6-folds decrease in wound area as compared to diabetic control and commercial product [94].

Simvastatin- (SV-) mesenchymal stem cell (MSC) NLCs in the form of scaffolds were evaluated against male albino Wistar diabetic rats. SV-MSC NLCs showed 55% burst release of SV within a period of 1 hour, and this value of SV release reached to 61.4% at the plateau level. The incorporation of SV-MSC NLCs into the scaffold delayed the initial burst release of SV on 24 h as compared to SV NLCs and maintained a more controlled drug release profile for 24 h by increasing the plateau level. Moreover, topical application of SV NLC-based scaffolds exhibited 3.1-folds, 1.19-folds, 2.5-folds, 1.02-folds, and 1.97-folds decrease in wound area as compared to diabetic control, placebo NLC scaffolds, free SV scaffolds, SV NLC scaffolds, and MSC scaffold-treated groups, respectively [71].

Alginate-based scaffolds have been also used successfully in treating DW. For instance, Karri et al. studied the *in vitro* drug release profile of alginate-collagen impregnated curcumin (ACIC) scaffolds and curcumin chitosan NPs (CCSNPs) in phosphate buffer saline pH 7.4. In addition, the wound healing potential of ACIC scaffolds and CCSNPs were investigated against diabetic rats having wound. *In vitro* drug release study demonstrated that in 3 h, CCSNPs showed a curcumin release of 21.71 ± 2.90%, whereas ACIC scaffolds exhibited curcumin release of 8.2 ± 3.43%. After 72 h 81.06 ± 3.32% of curcumin was released from CCSNPs while 56.24 ± 4.05% of curcumin was released from ACIC scaffolds. This indicated that ACIC scaffolds showed better sustained release of curcumin as compared to CCSNPs. *In vivo* wound healing study exhibited about 2.1-folds and 1.5-folds increase in wound contraction as compared to control (sterile gauze) and placebo scaffold-treated groups [95].

When the potential of graphene oxide-sodium alginate-polyhydroxy butyrate-scaffolds loaded with curcumin and *Gymnema sylvestre* (GS) (GO-PHB-SA-CUR&GS) was studied against DW in diabetic patients, it was found that topical application of GO-PHB-SA-CUR&GS scaffolds accelerated DW healing within 14 days by promoting collagen deposition, cell migration, and proliferation. In addition, GO-PHB-SA-CUR&GS scaffold-treated groups exhibited 1.18-folds, 1.01-folds, and 1.02-folds increase in cell viability as compared to diabetic control, GO-PHB-SA, and PHB-SA-treated groups. This indicated that GO-PHB-SA-CUR and GS-based scaffolds are cytocompatible, safe for topical use, and showed excellent effect against DW [96].

One of the studies on the effect of cellulose acetate-gelatin-based nanofibrous dressings loaded with berberine against DW in STZ-induced male Wistar diabetic rats was evaluated. The results indicated that the topical application of cellulose acetate-gelatin-based nanofibrous dressings loaded with berberine exhibited 1.23-folds and 3.01-folds increase in collagen density as compared to cellulose acetate-gelatin alone and negative control groups. In addition, cellulose acetate-gelatin-based nanofibrous dressings loaded with berberine-treated groups exhibited 2.12-folds and 1.63-folds increase in angiogenesis as compared to negative control and cellulose acetate-gelatin alone-treated groups [78]. Some of the studies wherein polysaccharide-based scaffolds have been utilized to treat DW are enlisted in Table 4.

#### 5.1.2. Proteins

Proteins are large complex molecules formed by the combination of several hundred to thousand units of amino acids. They are obtained from animals, plants, and marine source. They are essential for maintaining the structure, functions, and regulation of tissues and organs. Moreover, proteins are used in scaffold fabrication due to their biodegradation, bioabsorption, and biocompatibility [113]. In addition, the primary components of fibrous proteins are collagen, keratin, fibronectin, vitronectin, and elastin. These components help in the cell proliferation, migration, and provide cell to cell and cell to matrix interaction that helps in healing the wound. Various proteins that have been used to fabricate scaffolds for DW include collagen, hyaluronic acid, and fibrinogen. These are discussed below [114].

Collagen is abundantly found in bone, tendon, and ligaments. It is considered as a biological macromolecule that helps in the formation of highly organized 3D architecture. It can accommodate any component due to its network like structure, hence, utilized in tissue engineering. Other features such as mesh-like structure, spongy nature, porosity, and surface adsorption properties make them unique carrier for fabricating scaffolds. The porous nature of collagen allows transport of oxygen at the site of injury, and sponge-based structure is helpful in absorbing exudates of wounds which are responsible for bacterial growth. The mesh-like network present in collagen causes sustained release of drugs for a prolonged period of time. Furthermore, collagen has biological resemblance with the native collagen which is already present in our body. So, it can act as a cell-based scaffold for tissue rejuvenation applications. In addition to this, when collagen sponge is loaded with therapeutic substances such as GFs and cytokines, then they accelerate fibroblast formation, allow proliferation of keratinocytes, and accelerate wound healing process [115].

Fibrinogen is a glycoprotein complex formed in the liver. It consists of three pairs of polypeptide chains named as A*α*, *Bβ*, and *γ*, with molecular masses of 66.2, 54.5, and 48.4 kDa, respectively. It is found in the blood and plays a crucial role in platelets aggregation and blood clotting. It is used in scaffold fabrication because it provides excellent surface for cell proliferation and cell attachment. Moreover, it has high affinity for GFs such as VEGF and fibroblast growth factor (FGF) which are essential for wound healing. In addition, it has nanometric fibrous structure and mimick ECM that helps in stabilization of wound and allows support of local cell migration [116, 117]. Furthermore, it also helps in angiogenesis and repairs the wounds [118].

Hyaluronic acid (HA) is a negatively charged disaccharide polymer that consists of repeating units of N-acetylglucosamine and D-glucuronic acid. HA is most preferred biomaterial for scaffolds manufacturing due to its massive potential in wound healing. It helps in maintaining haemostasis by binding to the fibrinogen and commence blood clotting pathway [119]. Moreover, it also helps in inflammatory cell migration as well as promotes cell infiltration by creating oedema. Furthermore, it dampens inflammatory response by inhibiting migration of neutrophils towards wound site. In addition, it allows migration of fibroblasts at the site of injury and also fills the gaps of the lately formed ECM by providing cushioning and structural organization. Moreover, it helps in angiogenesis by stimulating matrix metalloproteinase and allows migration or proliferation of keratinocytes which help in wound healing. It also contributes in treating normal and pathological scarring by acting on remodelling phase [120]. Various protein-based scaffolds used so far to treat DW are discussed in the subsequent sections.

The activity of collagen binding domain-VEGF (CBD-VEGF) scaffolds was checked against DW in STZ-induced male SD diabetic rats. The CBD-VEGF-loaded collagen scaffold was found to exhibit 1.05-folds and 1.11-folds increase in wound closure within 14 days as compared to native VEGF- and PBS-treated groups. In addition, CBD-VEGF-loaded collagen scaffolds exhibited 3.6-folds and 1.8-folds increase in blood vessel density as compared to VEGF alone and phosphate buffer saline- (PBS-) treated groups [121].

Jiang et al. studied the effect of adipose-derived stromal vascular fraction cell- (ADSVF-) based collagen scaffolds against DW in STZ-induced domestic diabetic female pigs. In this study, percentage wound healing, blood vessel density, and VEGF levels of ADSVF-based collagen scaffold-treated groups were evaluated. The results were compared with diabetic control, SVFs, and collagen scaffolds alone-treated groups. The results revealed that the ADSVF-based collagen scaffold-treated groups exhibited 1.25-folds, 1.4-folds, and 1.6-folds increase in percentage wound healing as compared to collagen scaffolds alone, SVFs, and diabetic control groups, respectively. Furthermore, ADSVF-based collagen scaffolds exhibited 1.39-folds, 1.79-folds, and 2.04-folds increase in blood vessel density as compared to collagen scaffolds alone, SVFs, and diabetic control groups, respectively. In addition, ADSVF-based collagen scaffolds exhibited 1.32-folds, 1.65-folds, and 1.80-folds increase in VEGF levels in comparison to collagen scaffolds alone, SVFs, and diabetic control groups, respectively [122].

Elliott et al. investigated the effect of peritoin (PN) and connective tissue GF (CCN2) collagen-based scaffolds against DW in db/db diabetic mice. The scaffolds were fabricated by electrospinning technique. The fabricated scaffolds were evaluated for wound healing, vascularization, and blood vessel density studies. The results revealed that on the 11^th^-day diabetic control, collagen, CCN2, and PN scaffold-treated groups showed wound size of 5.8, 5, 3, and 4 mm, respectively. Furthermore, it was observed from the study that CCN2-treated groups showed maximum vascularization as compared to other groups. The angiogenesis study revealed that CCN2 scaffolds exhibited 1.75-folds, 3.5-folds, and 4.66-folds increase in vessel density as compared to PN, collagen, and diabetic control groups, respectively [123].

A study was carried order to evaluate the effect of B-cell lymphoma-2- (Bcl-2-) modified adipose-derived stem cell (ADSC) collagen-based scaffolds against DW in STZ-induced db/db diabetic mice. It was found to exhibit 1-fold, 1.9-folds, and 1.72-folds increase in wound healing rate as compared to ADSC scaffolds, diabetic control, and placebo scaffolds, respectively. The angiogenesis study revealed that the blood vessel density of Bcl-2-ADSC scaffolds was found 1.42-folds, 10.2-folds, and 6.8-folds higher than that of ADC scaffolds, diabetic control, and placebo scaffolds, respectively [124].

Karri et al. explored the potential of collagen-chitosan scaffolds loaded with curcumin and chitosan NPs (Cur-CSNPs) against DW in STZ-induced male Wistar diabetic rats. In this study, percentage wound closure of collagen-chitosan scaffolds loaded with Cur-CSNPs was evaluated. The results were compared with diabetic control and placebo scaffold-treated groups. The results revealed that the collagen-chitosan scaffolds loaded with Cur-CSNPs exhibited 2.19-folds and 1.59-folds increase in wound closure as compared to diabetic control and placebo scaffold-treated groups. In addition, collagen-chitosan scaffolds loaded with Cur-CSNPs accelerated DW healing within 15 days by promoting collagen deposition, granulation tissue formation, epithelialization, anti-inflammatory, and antioxidant effect at the wound site as compared to other groups [125].

Anti-inflammatory effect of VEGF-SDF-1*α*-loaded collagen scaffolds was studied in STZ-induced male SD diabetic rats. Topical application of VEGF-SDF-1*α*-loaded collagen scaffolds accelerated DW healing by promoting collagen deposition, reepithelization, and angiogenesis at the wound site. In addition, VEGF-SDF-1*α*-loaded collagen scaffold-treated groups showed 2-folds and 3.57-folds decrease in IL-1*β* and TNF-*α* levels as compared to placebo scaffold-treated groups [126].

The effect of osteopontin- (OPN-) treated autologous circulating angiogenic cell-based collagen scaffolds (CACs-OPN-col scaffolds) was tested against ulcer created in alloxan-induced diabetic rabbits. The developed scaffolds exhibit 1.12-folds, 1.31-folds, and 1.55-folds increase in percentage wound closure as compared to CACs-collagen scaffold without OPN treatment, collagen scaffolds alone, and diabetic control, respectively [127].

In one of the studies, Wan et al. examined the effect of the combination of silver and platelet-derived GF-BB (PDGF-BB) gelatin-based scaffolds against DW in STZ-induced female C57BL/6JNju DIO type II diabetic mice. The scaffolds were fabricated by a 3D bioprinter. The scaffolds were tested for biocompatibility and angiogenesis. The angiogenesis study revealed that combination of silver and PDGF-BB scaffolds exhibited 2-folds, 2.28-folds, and 1.23-folds increase in angiogenesis as compared to placebo scaffolds, silver/scaffold, and PDGF-BB/scaffolds, respectively. The biocompatibility study showed that the relative growth rate of human leukemia (HL) 60 cells for silver/PDGF-BB scaffolds (0.5 mg/mL) and silver/PDGF-BB scaffolds (1 mg/mL) was found to be 96.05 ± 5.21% and 95.12 ± 4.28% on day 3 and 94.97 ± 3.33% and 93.01 ± 5.35% on day 5, respectively, indicating biocompatibility of silver/PDGF-BB scaffolds [128].

Yang et al. investigated the effect of coadministration of gelatin (G), collagen (Co), *Lithospermi radix* (LR), and curcumin (C) (G/Co/LR/C) scaffolds against STZ-induced DW on male SD diabetic rats. The scaffolds were fabricated by electrospinning technique. The fabricated scaffolds were tested for percentage wound recovery and collagen deposition studies. The results of *in vivo* studies revealed that G/Co/LR/C scaffolds exhibited 1.09-folds, 1.04-folds, 1.05-folds, 1.12-folds, and 1.07 folds increase in wound healing rate as compared to G/Co, G/Co/LR, marketed formulation (Comfeel®), and gauze (control)-treated groups, respectively. The results of collagen production studies showed that G/Co/LR/C scaffolds exhibited 1.4-folds, 1.2-folds, 1.1-folds, 1.3-folds, and 1.4-folds increase in collagen content as compared to G/Co/C, G/Co/LR, G/Co, Comfeel® (marketed scaffold) and gauze (control)-treated groups, respectively [129].

Fibrin-based scaffolds have been also used to treat DW; for instance, Losi et al. investigated the wound healing effect of fibrin-based scaffolds loaded with VEGF-bFGF in STZ-induced male db/db diabetic mice. Topical application of fibrin-based scaffolds loaded with VEGF-bFGF accelerated DW healing by promoting reepithelization, angiogenesis, collagen deposition, and granulation tissue formation at the wound site. *In vivo* wound healing study revealed that the fibrin-based scaffolds loaded with VEGF-bFGF-treated groups exhibited 2-folds, 5-folds, and 8-folds increase in wound as compared to scaffolds loaded with GFs/NPs, placebo scaffolds, and control scaffolds, respectively [130]. In another study, Losi et al. examined the wound closure potential of fibrin/poly(ether)urethane-based (FPU) scaffold loaded with platelet lysate against DW in STZ-induced male db/db diabetic mice. The results revealed that the FPU scaffold loaded with platelet lysate-treated groups exhibited 1.5-folds, 2.85-folds, and 3.87-folds increase in percentage wound closure as compared to FB-GF scaffolds, FB scaffolds, and polyurethane film (Mepore®)-treated groups, respectively [131].

HA-based scaffolds have been also utilized to treat DW. For instance, Shin et al. investigated the wound closure effect of HA and PLGA scaffolds loaded with epigallocatechin-3-O-gallate against DW in STZ-induced male Sprague Dawley (SD) diabetic rats. The results revealed that HA- and PLGA-based scaffold-loaded epigallocatechin-3-O-gallate-treated groups exhibited 4.6-folds, 4.4-folds, 3.7-folds, and 2.2-folds decrease in wound area as compared to control, PLGA, HA/PLGA, and Rapiderm® (marketed formulation)-treated groups, respectively [132].

In one of the studies, Angelis et al. explored the wound closure and reepithelization potential of HA scaffold-loaded platelet-rich plasma against (HA + PRP) DW in DFU patients. The results revealed that HA + PR-loaded scaffold-treated groups exhibited 2-folds and 1.1-folds increase in wound closure and reepithelialization as compared to HA alone-treated groups [133].

Besides, collagen, fibrin, and hyaluronic acid keratin-based scaffolds are also explored to treat DW. In one of the studies, Konop et al. explored the wound healing potential of casomorphin-keratin-based scaffolds (CKS) against DW in STZ-induced C57BL/6J diabetic mice. Topical application of CKS scaffolds accelerated DW healing by promoting reepithelization, macrophage infiltration, and reducing microhemorrhage at the wound site. The results also revealed that the CKS-treated groups exhibited 1.15-folds increase in wound healing rate as compared to control groups (citrate buffer treatment) [134].

The protein-based scaffolds explored so far to treat DW are discussed below in Table 5.

### 5.2. Synthetic Polymers

The first synthetic polymer used as a suture was polyglycolide, which came under the trade name Dexon. The other synthetic polymers which are utilized as implantable materials include poly (tetrafluoroethylene), polyethylene glycol, silicone, copolymers of PLA, polyurethanes, and poly (glycolic acid) (PGA). They are used in tissue engineering because of their inertness and biocompatibility. In addition, they are cheaper and easy to fabricate [87]. Various synthetic polymers used to fabricate scaffolds are discussed below.

#### 5.2.1. Polyesters

Polyesters are synthetic polymers that consist of chains of ester functional groups in their main chain. They are generally prepared by the polycondensation of 2,3-bis(4-hydroxyphenyl)-5-azaquinoxaline with aromatic and aliphatic dicarboxylic dichlorides [153]. They are used in tissue engineering because of their biocompatibility and biodegreadibility [154]. Various polyesters which have been used in tissue engineering are poly (L-lactic acid) (PLLA), poly (glycolic acid), poly (trimethylene carbonate) (PTMC), polycaprolactone, polyvinyl alcohol, and poly (propylene fumarate) (PPF) [154]. Among them, PLA, PVA, and polycaprolactone are mostly used in tailoring scaffolds for DW.

PLA and PGA are widely acceptable as polymers for scaffold fabrication. They offer excellent tissue rejuvenation because they are easy to process, and from many years, they have been utilized as an implantable material in medicine. Moreover, PLA and PLGA also promote lactate supply at the wound site which further promotes angiogenesis and recruit endothelial progenitor cells and accelerates wound healing [155, 156]. In addition, they are considered as good drug carrier due to their prolonged retention time at the injury site and ability to release the drug for a desired period that facilitates effective wound healing process [155, 156].

Polycaprolactone is a linear aliphatic semicrystalline and hydrophobic polymer. It has been extensively utilized in wound healing. Polycaprolactone deficits bioactivity and has high degradation rate that can be modified by varying crystallinity, molecular weight, or modification in the structure by using hydrophilic creamics and polyethylene glycol or by making copolymers with PGA and PLA. Further, coating of polycaprolactone with gelatin, collagen, and calcium phosphate helps in cell migration, cell proliferation, and endothelial cell adhesion [157]. In addition, it has desirable properties such as stability, easy processing, and has been approved from United States Food and Drug Administration (USFDA) as materials for sutures and wound dressings [158].

PPF is a biodegredable synthetic polymer and extensively used in wound healing due to its immense biomedical applications such as tunable degradation, nontoxicity, controllable mechanical properties, and biocompatibility [159]. Moreover, it is used along with ceramic particles such as calcium phosphate and calcium carbonate that help in the replacement of cancellous bones [160]. In addition, PPF also helps in wound healing by showing anti-inflammatory action and promotes reepithelization at the wound site [161].

PEG is a hydrophilic polymer that is prepared by the addition of ethylene oxide to the diethylene glycol. It is used in tissue engineering due to its inherent advantages such as flexibility, nonimmunogenicity, nontoxicity, and biocompatibility. Moreover, it can be bonded along with epidermal growth factor (EGF) for wound healing [162]. In addition, it is bonded along with PLGA and chitosan for enhancing its crystallinity and thermal stability. The use of PEG as wound dressing material in DW helps in reducing scar formation and promotes collagen deposition at the wound site [163]. It is also used in the preparation of hydrogel-based dressings in combination with HA and adipose-derived stem cells, which support cell viability both *in vitro* and *in vivo*. It also acts as a temporary hydrogel and prevents wound contraction as well as promote angiogenesis at the wound site [164, 165]. Various studies wherein polyester-based synthetic polymers have been used to treat DW are discussed below.

Lv et al. investigated the effect of silicate-based bioceramic particles (NAG)/poly (caprolactone) (PCL)/gelatin- (Ge-) based nanofibrous composite scaffold against DW healing in STZ-induced female C57BL/6 diabetic mice. The scaffolds were prepared by electrospinning technique and tested for cell adhesion, human umbical vascular endothelial cell (HUVEC) migration, histocompatibility, and wound healing studies. The results of cell adhesion study showed that NAG/PCL/Ge-based scaffolds exhibited 1.98-folds increase in cell adhesion as compared to PCL scaffold-treated groups. To evaluate cell migration, HaCaT cell line was used. The results of cell line study revealed that NAG/PCL/Ge-based scaffolds exhibited 1.79-folds and 1.70-folds increase in cell migration as compared to diabetic control and PCL-treated groups, respectively. The results of wound healing study showed that NAG/PCL/Ge-based scaffolds exhibited 1.14-folds and 1.36-folds increase in wound closure as compared to PCL and diabetic control-treated groups. The results of histocompatibility studies revealed that the thickness of epidermis in NAG/PCL/Ge-based scaffold-treated groups was found to be 1.8-folds and 2.45-folds higher than that of PCL and diabetic control groups, respectively. Furthermore, it was observed from the study NAG/PCL/Ge-based scaffold-treated groups exhibited 1.43-folds and 1.67-folds increase in collagen deposition as compared to PCL-treated and diabetic control-treated groups, respectively [166].

A study was carried out by Cheng et al. to evaluate reepithelization and angiogenesis activity of bone marrow mesenchymal stem cell- (BMSC-) based radially aligned scaffolds (RAS + BMSCs) and BMSC-based vertically aligned scaffolds (VAS + BMSCs) against DW in STZ-induced male TALLYHO T2D mice. It is an inbred polygenic mice model that is generated to inducing T2DM and moderate obesity in the mice. The developed scaffolds were found to exhibit 2.2-folds, 2.5-folds, and 1.05-folds increase in reepithelization rate as compared to diabetic control, VAS, and combination of VAS and BMSC-treated groups, respectively. In addition, combination of RAS and BMSC-treated groups exhibited 1.2-folds, 1.4-folds, and 1.7-folds increase in angiogenesis in comparison to mice treated with combination of VAS and BMSCs, VAS alone, and RAS alone-treated groups, respectively [167].

Ilomuanya et al. investigated the reepithelization and wound closure effect of PLA-valsartan-based hydrogel-based scaffolds against DW in STZ-induced male diabetic rats and compared its efficacy with neomycin-based dressings. The results of reepithelization study revealed that the PLA-valsartan-based hydrogel scaffold-treated groups showed 1.43-folds increase in reepithelialization rate as compared to neomycin-based dressings. In addition, PLA-valsartan-based hydrogel scaffold-treated groups exhibited 2.5-folds increase in wound closure as compared to neomycin-based dressing-treated groups [168].

Lee et al. evaluated the epidermis thickness and wound closure effect of PLGA-PDGF-based scaffolds against DW in STZ-induced male diabetic SD rats and compared its results with phosphate buffer saline (PBS)/gentamicin/PLGA and PLGA blended with gentamicin. The results showed that PLGA-PDGF-based scaffold-treated groups exhibited 4.7-folds and 5.2-folds increase in epidermis thickness within 2 weeks as compared to phosphate buffer saline (PBS)/antibiotics/PLGA and PLGA blended with gentamicin-treated groups. In addition, PLGA-PDGF-based scaffold-treated groups exhibited 1.14-folds and 1.7-folds increase in wound closure as compared to phosphate buffer saline (PBS)/gentamicin/PLGA and PLGA blended with gentamicin only [169].

A study was carried out by Han et al. to evaluate the effect of 30 asiatic acid- (AA-) embedded aligned porous PLLA fibrous scaffold (AA-PL) against male diabetic C57BL/6J mice. The results of the wound healing study revealed that 30 AA-PL scaffolds exhibited 1.93-folds, 1.47-folds, and 1.03-folds increase in wound closure as compared to control, PL, and 10 AA-PL scaffolds, respectively. The results of the reepithelialization study showed that the 30 AA-PL scaffolds showed 1.85-folds, 1.47-folds, and 1.21- folds increase in reepithelialization rate as compared to control, PL, and 10 AA-PL scaffolds, respectively. The results of the collagen deposition study revealed that 30 AA-PL scaffold-treated mice showed 1.54-folds, 1.26-folds, and 1.08-folds increase in collagen deposition as compared to control, PL, and 10 AA-PL scaffold-treated mice, respectively [170].

The potential of core shell insulin-loaded PLGA-based nanofibrous scaffolds were checked against DW in STZ-induced SD diabetic rats. *In vitro* drug release study showed that core shell insulin-loaded PLGA-based nanofibrous scaffolds released drug at steady rate for 28 days. The release rate was found 12.8 ± 4.8 mU/mL, 10.7 ± 1.1 mU/mL, and 15.7 ± 1.2 mU/mL on days 1, 3, and 21, respectively. Afterwards, the concentration of drug gradually decreased to 8.5 ± 1.9 mU/mL on the 28^th^ day. This indicated that core shell insulin-loaded PLGA-based nanofibrous scaffolds prolonged the release of insulin in a sustained manner that increased the retention of drug at the wound site for a longer period of time. Wound closure study revealed that core shell insulin-loaded PLGA-based nanofibrous scaffolds showed 3.01-folds and 3.16-folds decrease in wound area within 14 days as compared to core PBS and shell PLGA and blend of insulin and PLGA. In addition, core shell insulin-loaded PLGA-based nanofibrous scaffolds exhibited 1.87-folds and 2.17-folds increase in collagen to glyceraldehyde 3-phosphate dehydrogenase (GAPDH) ratio in comparison to core PBS and shell PLGA and blend of insulin and PLGA [171].

In one of the studies, Chen et al. studied the effect of PEG–desferrioxamine (DFO) hydrogel-based scaffolds against DW in STZ-induced male SD diabetic rats. The results revealed that PEG-DFO hydrogel-based scaffold-treated groups exhibited 1.2-folds and 1.5-folds increase in length of epidermis as compared to placebo hydrogel and diabetic control groups. *In vivo* wound healing study revealed that PEG-DFO hydrogel-based scaffold-treated groups exhibited 4-folds and 7-folds increase in wound closure as compared to placebo hydrogel and diabetic control groups [172].

#### 5.2.2. Polyvinyl Alcohol

Polyvinyl alcohol (PVA) is a synthetic polymer formed by the hydrolysis of vinyl acetate. It is used mostly in food industries for packaging, paper industries for the production of paper as well as used in medical devices. The utilization of PVA in medical applications are increasing at immense rate due to their nontoxicity, biocompatibility, bioadhesive characteristics, swelling properties, and noncarcinogenic nature. Owing to their enormous potential of PVA is used in replacement of cartilages, soft contact lenses, eye drops, and tissue adhesion barriers [59]. In addition, they are used in the fabrication of wound dressings because of their biodegradable nature, exudate absorbing capacity, and fiber formability. Furthermore, fibers of PVA have been extensively used in tissue rejuvenation as they provide help in cell proliferation and allow cell adhesion and breathability for cellular growth [173]. Owing to these advantages, PVA-based scaffolds are used in the treatment of DW. Some of the studies wherein PVA-based scaffolds have been used to treat DW are discussed below.

A study was carried out to investigate the effect of brown algae-derived polysaccharide (BAP)-PVA-based scaffolds against DW in STZ-induced male C57 diabetic mice. Topical application of BAP-PVA-based scaffolds accelerated DW healing within 12 days by promoting neo-vascularization, anti-inflammatory action, and cell proliferation at the site of injury. In addition, BAP-PVA-based scaffold-treated group exhibited 1.05-folds and 1.17-folds increase in wound closure in comparison to PVA alone treated mice and diabetic control groups [174].

The activity of nanofibrous mats prepared from PVA, chitosan, and zinc were checked against DW in STZ-induced male SD diabetic rats. Nanofibrous mats prepared from PVA, chitosan, and zinc were found to exhibit about 1.8-folds increase in wound closure as compared to chitosan-PVA alone-treated group. The enhanced activity of nanofibrous mats prepared from PVA, chitosan, and zinc were attributed to its increased collagen deposition and antibacterial effect at the site of injury [175].

In another study, the effect of chitosan-PVA based nanofibrous scaffolds was studied against DW in STZ-induced male Wistar diabetic rats. It was found that the topical application of chitosan-PVA-based nanofibrous scaffolds accelerated DW healing within 14 days by promoting antibacterial action and collagen deposition at the site of injury. On the 14^th^ day, chitosan-PVA-based nanofibrous scaffold-treated groups showed reduction in average wound area of diabetic rats by 22-folds as compared to diabetic control group [176].

The activity of scaffolds prepared from CS and PVA was checked against DW in STZ-induced male SD diabetic rats. Topical application of prepared scaffolds accelerated DW healing within 15 days by showing 2-folds and 1.1-folds decrease in wound area as compared to diabetic control and PCL-PVA-CS-based scaffold-treated groups. In addition, it promoted antibacterial action, granulation tissue formation, epidermal cell regeneration, and dermal tissue proliferation at the site of injury that helped in DW healing [177].

In one of the studies, wound healing potential of PVA nanoscaffolds loaded with propolis NPs was checked against DW in STZ-induced male Swiss diabetic mice. The results revealed that PVA nanoscaffolds loaded with propolis NPs exhibited 2.3-folds, 1.2-folds, and 1.3-folds increase in wound closure in comparison to negative control (no treatment), PVA alone, and positive control (allantoin-based treatment) groups [178].

In another study, angiogenesis effect of PLA-PVA scaffolds loaded with connective tissue growth factor (CTGF) was evaluated against DW in chicken chorioallantoic membrane (CAM). The results showed that PLA-PVA scaffolds loaded with CTGF exhibited 1.2-folds and 1.3-folds increase in angiogenesis as compared to placebo scaffolds and normal control group [179].

The wound healing activity of PVA-CS nanofibers was checked against DW in STZ-induced male Wistar diabetic rats. Topical application of prepared scaffolds accelerated DW healing by promoting antibacterial action at the wound site. *In vivo* wound healing study revealed that PVA-CS nanofibers exhibited increase in wound closure by 1.3-folds in comparison to diabetic control group [180]. Lists of various synthetic polymers explored to treat DW are enumerated in the Table 6.

## 6. Scaffold Fabrication Techniques

Scaffolds are 3D polymeric structures which supports tissue rejuvenation upon topical application at the wound site. They can be fabricated by using conventional and rapid protyping (RP) techniques. In conventional scaffold fabrication techniques, there is construction of porous polymeric structures, i.e., substrates for cell adhesion, but does not provide tunable macroscale and microscale complex structures. RP is an advanced form of conventional technique that provides a plethora of potential opportunities in the field of tissue engineering. This technique provides independent control over macroscale and microscale features that helps in the formation of multicellular structures required for complex tissue functions. In addition, this technique also helps in fabrication of three-dimensional vascular beds needed for massive tissue formation. With the advancement the technology, researchers have combined clinical imaging data, and 3D fabrication techniques can provide the possibility of production of customized scaffolds as well as mass production of the scaffold designs [199, 200]. The classification of scaffolds on the basis of fabrication are given in Table 7.

## 7. Miscellaneous

Chu et al. investigated the potential of MSC-acellular dermal matrix (ADM) scaffolds for DW healing in STZ-induced male ICR diabetic mice. The fabricated scaffolds were tested for wound healing, vessel density, and epidermal thickness studies. The wound healing studies demonstrated that MSC-ADM scaffolds showed 1.1-folds and 1.07-folds increase in wound closure as compared to control and ADM scaffold-treated groups, respectively. The angiogenesis study revealed that MSC-ADM scaffolds exhibited 1.52-folds and 2.4-folds increase in vessel density. Furthermore, MSC-ADM scaffolds exhibited 1.6-folds and 2.7-folds increase in epidermal thickness as compared to control and ADM scaffold-treated groups, respectively [201].

A study was carried out to investigate the effect of ADM-reduced graphene oxide (RGO)-MSCs composite scaffolds against DW in STZ-induced male ICR diabetic mice. The results revealed that ADM-RGO-MSC composite scaffolds exhibited 1.05-folds, 1.5-folds, and 1.62-folds increase in regenerative collagen percentage within 28 days as compared to ADM-GO-MSCs, ADM-MSCs and diabetic control groups, respectively. The cell migration studies showed that ADM-RGO-MSCs composite scaffolds exhibited 1.08-folds and 1.87-folds increase in cell migration as compared to ADM-GO-MSC- and ADM-MSC-treated groups. The results of angiogenesis study revealed that ADM-RGO-MSC composite scaffold-treated groups showed 1.09-folds, 1.3-folds, and 2.05-folds increase in vascular density within 4 weeks as that of ADM-GO-MSCs, ADM-MSCs, and diabetic control-treated groups, respectively. In addition, ADM-RGO-MSC composite scaffold-treated groups showed 1.12-folds, 1.38-folds, and 1.66-folds increase in granulation thickness within 28 days in comparison to ADM-GO-MSCs, ADM-MSCs, and control-treated groups, respectively. The wound healing studies revealed that ADM-RGO-MSC composite scaffolds exhibited 1.11-folds, 1.66-folds, and 2.08-folds decrease in wound area within 28 days as compared to ADM-GO-MSCs, ADM-MSCs, and diabetic control-treated groups, respectively [202].

In one of the studies, effect of wingless and hUCMSCs (Int-1 (Wnt) signaling pathway agonist (Wnt3a)-human umbilical cord MSCs) scaffolds and Wnt signaling pathway antagonist (sFRP3)-hUCMSC scaffolds were checked against DW in STZ-induced male SD diabetic rats. The results revealed that Wnt3a-hUCMSCs scaffold-treated groups exhibited 1.42-folds and 1.25-folds increase in wound closure as compared to sFRP3-hUCMSC scaffolds and diabetic control groups. Furthermore, Wnt3a-hUCMSC scaffold-treated groups showed equivalent wound healing as compared to normal control group. In addition, Wnt3a-hUCMSC scaffold-treated groups exhibited 1.36-folds, 2-folds, and 3.3-folds increase in skin appendage regeneration as compared to normal control, diabetic control, and sFRP3-hUCMSC scaffold-treated groups, respectively. Moreover, Wnt3a-hUCMSC scaffold-treated groups exhibited 3.6-folds and 8-folds increase in hUCMSC differentiation as compared to normal control and sFRP3-hUCMSC scaffold-treated groups. The CCK8 staining study revealed that Wnt3a-hUCMSC scaffold-treated groups exhibited 1.25-folds and 1.87-folds increase in cellular viability in comparison to normal control and sFRP3-hUCMSC scaffold-treated groups [203].

Vijayan et al. studied the *in vitro* drug release effect of dual GF (VEGF, bFGF)-based nanofibrous scaffolds in phosphate buffer saline pH 7.4. In addition, the wound closure and cell viability rate of GF-NPs-nanofibrous (NF) scaffolds was evaluated against DW in STZ-induced Swiss albino diabetic mice. *In vitro* drug release studies revealed that the developed nanofibrous scaffolds exhibited 1.2-folds, 1.16-folds, and 1.08-folds increase in release of VEGF in a sustained manner as compared to bFGF scaffolds in 50 h, 190 h, and 270 h, respectively. The wound closure study showed that GFs-NPs-NF scaffold-treated groups exhibited 1.18-folds, 1.7-folds, and 1.94-folds increase in percentage wound closure as compared to GF-NPs, NF alone, and control groups, respectively. Furthermore, GFs-NPs-NF scaffold-treated groups exhibited 1.3-folds, 1.25-folds, 1.2-folds, and 1.6-folds increase in cell survival rate as compared to NF alone, GF-NPs, GFs alone, and control groups, respectively [204].

In one of the studies, potential of human amniotic membrane (HAM) scaffolds was studied against DW in STZ-induced male adult Wistar diabetic rats. The results revealed that HAM scaffold-treated groups exhibited 1.5-folds and 1.7-folds increase in percentage wound closure as compared to HAM matrix and normal control groups. Furthermore, HAM scaffold-treated groups exhibited 1.4-folds increase in volume of epidermis as compared to HAM matrix and normal control groups. The results of collagen deposition study revealed that HAM scaffold-treated groups showed 1.05-folds and 12-folds increase in collagen deposition within 21 days as compared to HAM matrix and normal control groups [205].

## 8. Miscellaneous Nondiabetic Wound Electroconductive Scaffolds

A wide variety of electrically active polymers and conductive doping agents have been utilized in the fabrication of electroconductive scaffolds because of their numerous favorable positive biological responses at cellular and tissue level such as wound healing, biocompatibility, proliferation, and tissue regeneration that make them as a suitable candidates as conductive scaffolds for tissue regeneration with promising results [206]. In addition, these scaffolds mimick the extracellular matrix (ECM) and, as well as provide electrical stimulation to the injured tissues that helps in the tissue rejuvenation [207, 208]. Looking at these potentials, various electroconductive scaffolds used for the management of different damaged tissue are depicted in the Table 8.

**Figure 3:**
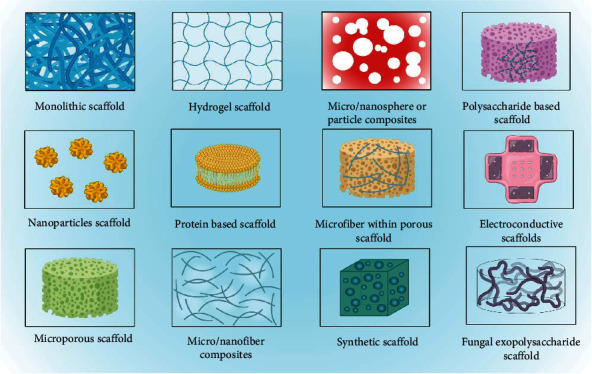
The pictorial representations of different type of scaffolds available for wound healing.

The pictorial representations of different type of scaffolds available for DW are given in Figure 3.

## 9. Conclusion and Future Perspectives

The market of scaffolds is growing rapidly across the globe. In 2020, its market size was valued at USD 1.1 billion that is expected to expand at a compound annual growth rate (CAGR) of 8.4% from 2021 to 2028 [209]. The scaffold market is basically divided into four major segments based on its end-use, technology, type of product, and type of application. On the basis of end-use, these have been basically, divided into three subsegments, i.e., cell attachment and migration, nutrients, and products diffusion, and cell phase behaviour modification. On the basis of technology, they are categorized as synthetic scaffolds, decellularized scaffolds, and 3D printed scaffolds, whereas, on the basis of type of product, these are divided into hydrogels, polymeric scaffolds, nanofiber-based scaffolds, and micropatterned surface microplate. Lastly, on the basis of application, these are divided into cancer cell research, stem cell research, regenerative medicine, tissue engineering, and cell-based assays.

The main research behind the rise in market size is attributed to the increase in the demand for 3D cellular scaffolds in biological studies and translational research. Some of the marketed scaffolds and patents are listed in Tables 9 and 10, respectively. The demand of 3D scaffolds is growing due to its efficiency in mimicking the *in vivo* physiological state for better presentation of disease-causing microenvironmental factors. Further, significant advancements in regenerative medicines and tissue engineering have increased the utilization of 3D bioprinting for organs and tissue reconstruction procedures.

In recent years, some of the companies such as CELLINK GLOBAL and its TRIANKLE consortium developed new personalized gelatin- and collagen-based implants with the application of 3D printing technology for ankle joints tissue regeneration [209]. Many other attempts have also been made to develop 3D scaffolds for treating cancer, and most recently, an attempt has been made by the company Ligandal, who developed a peptide scaffold as a prospective antidote and vaccine against corona virus disease-19 (COVID-19). The hydrogel-based scaffolds have given the largest revenue share of 14.2% in 2020 [209, 210]. Further, the microfabrication of hydrogels is expected to further increase the demand of hydrogel-based scaffolds. In March 2021, Cultrex UltiMatrix Reduced GF Basement Membrane Extract (RGF BME) was introduced by company Bio-Techne Corporation for culturing pluripotent stem cells and organoids, which is an advanced matrix hydrogel with optimized ECM protein composition, increased total protein content, and improved tensile strength for various applications such as personalized medicine, drug discovery research, and regenerative medicine.

The nanofiber-based scaffold market is also increasing with CAGR of 9.8% as of report of 2020. Despite these significant advancements in the field of scaffolds, the research on scaffolds for DW remains a challenge. There are many reports related to development of natural/synthetic polymer-based scaffolds for DW in *in vitro*/animal models. However, their translation to clinical application is facing still some hurdles.

The implantation of scaffolds is a challenging task as it is related to tissue engineering and tissue regeneration. Hence, the mechanical properties of scaffolds as well as their implantation in a proper way at the target site is really important. To achieve a successful response and efficacy among the patients suffering from DW or, severely diseased tissues or, organs, there is a much-needed requirement of proper coordination between medical surgeons and biomaterial scientists who can implant as well as evaluate the performance of scaffolds. It is the biomaterial scientist who can better select the material for designing scaffold. For example, the mechanical property of the scaffold can be enhanced by suturing. However, the materials like collagen, which are known for their softness and porosity, are unable to endure fixation with suture due to their low tearing strength. In such cases, reasonable fibers are more preferred. For instance, nonwoven PGA fabrics and beta-caprolactone homopolymer have been extensively used to fabricate scaffolds and reinforced by suturing. But due to high porosity of the PGA fabrics, the entrapment of cells inside them is poor. Similarly, beta-caprolactone homopolymer have poor resorption rate.

Similar to the challenges related to biomaterials used for scaffolds, the process of manufacturing scaffolds also has an impact on its quality as well as performance. In recent years, electrospinning technique has been widely reported for fabrication of scaffolds. It produces nanofibers from polymer solution using simple procedure. However, the nanofiber-based sheets offer too small pore sizes for allowing cell seeding. The solid free protocol technique has also been utilized for scaffold fabrication but it needs extensive and sophisticated instruments for fabrication. Hence, it is important to look forward for amalgamation of biomaterial science and tissue engineering.

Hence, there is a requirement to understand the factors that implement their clinical translation and find out the possibilities to overcome these challenges. Furthermore, based on the complexity of wound healing, various bioactive agents with specific release profile can be tailored into scaffolds. In addition, drug-polymer compatibility for designing scaffolds for DW should also be considered. Nevertheless, scaffolds have appeared as boon in the area of DW owing to their advantages over conventional dressings. In the future, it is expected that scaffolds will utilize theranostic materials that would offer interactive as well as bioactive means along with therapeutic and diagnostic function in a single unit. Further, the incorporation of biomarkers in scaffolds would enable the monitoring of wound healing process.

## Figures and Tables

**Table 1:** Advantages and disadvantages of stimuli-responsive wound dressings.

Stimuli	Advantages	Disadvantages	References
Temperature	(i) Antibacterial action (ii) Quick response to temperature change	(i) Dressings should be intact with the wound	[34]
pH	(i) Ease of fabrication (ii) Simple composition	(i) High pH is required for drug release	
Glucose	(i) Control blood glucose level and heal the wounds	(i) Release speed of insulin cannot be well controlled	
Reactive oxygen species (ROS)	(i) Easy to apply (ii) Simple composition (iii) Regulate inflammatory process	(i) Enter the systemic circulation	
Near-infrared light (NIR)	(i) Improve microcirculation (ii) Promote angiogenesis	(i) Temperature increase needs to be controlled carefully	

**Table 2:** Studies on nanocarriers implemented in dressings/scaffolds.

Drug	Nanocarrier	Animal model	Results	Ref
*Scaffolds*				
Simvastatin	NLCs	Albino Wistar rats	Exhibited 1.2-folds and 2.7-folds decrease in wound area as compared to placebo scaffolds and free simvastatin-treated groups	[38]
GF	Polylactic glycolide acid NPs	db/db mice	Exhibited 1.05-folds, 1.53-folds, and 1.48-folds increase in wound contraction as compared to PLGA-NPs, control, and VEGF alone-treated groups, respectively	[39]
Silver	NPs	Rabbits	Accelerated wound healing by promoting antibacterial action, collagen deposition, and fibroblast migration at the site of injury	[40, 41]
Konjac glucomannan, keratin and Avena sativa extract	Hydrogel	Wistar rats	Hydrogel scaffolds showed 2.08-folds increase in antioxidant activity as compared to diabetic control group	[42]
Glucophage	NPs	SD rats	Exhibited about decreases in wound area by 3.5-folds within 14 days in comparison to gauze sponge treated groups	[43]
Psyllium seed husk polysaccharide, keratin, and 1% morin	Hydrogel	Wistar diabetic rat	Exhibited 2.4-folds, 1.07-folds, and 2.15-folds increase in wound closure as compared to diabetic control, PSH, KER, and 0.5% MOR coloaded scaffolds and combination of PSH and KER scaffold-treated groups, respectively	[44]
*Dressings*				
Chemokine	Gelatin hydrogel	ICR mice	Exhibited 1.8-folds faster wound contraction as compared to gelatin hydrogel alone	[45]
Polyvinyl alcohol	Hydrogel	db/db mice	Accelerated DW healing in 16 days by promoting angiogenesis, granulation tissue formation, and releasing nitric oxide at the site of injury	[46]
Polymerized ionic liquids	Hydrogel	Male Kunming mice	Promoted DW healing in 14 days by showing migration and proliferation of fibroblast cells at the site of injury	[47]
Fibroblast GF	Hydrogel	SD rats	Accelerated DW healing by showing increase in Ki67 expression, neovascularization, and epithelialization at the site of injury	[48]
Combined reactive oxygen species	Cerium oxide NPs	SD rats	Showed DW healing in 14 days by promoting angiogenesis, collagen deposition, and neovascularization at the site of injury	[49]

**Table 3:** Polymers used in fabrication of scaffolds.

Polymer	Structure	Advantages	Disadvantages
*Polysaccharides*			
1. Alginate It is obtained from brown seaweeds [50] Chemically, it is anionic in nature and consists of copolymers of D-mannuronic acid (M monomer) and L-guluronic acid (G monomer) [51]	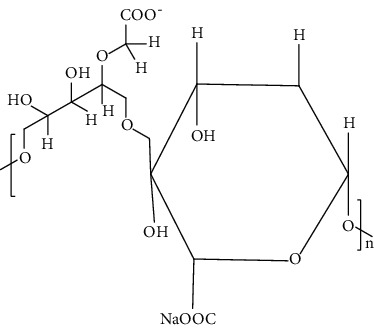	(i) Biocompatibility (ii) Ease of gelation (iii) Low toxicity (iv) Bioadhesive (v) Neovascularization	(i) Limited control of mechanical properties (ii) Ion-leaching leading to instability
2. Chitosan It is obtained from the walls of fungi and shrimps and can be synthesized synthetically Chemically, it is *β*-(1/4)-linked D-glucosamine and N-acetyl-D glucosamine-based cationic linear polymer [52]	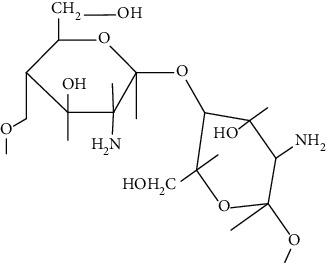	(i) Good biocompatibility (ii) Biodegradability (iii) Cellular binding capability (iv) Antimicrobial activity (v) Wound healing effect	(i) Low mechanical strength (ii) Low-temperature response rate
3. Cellulose It is obtained from plants, oomycetes, and algae Chemically, it consists of *β* (1→4)-linked D-glucose units [53].	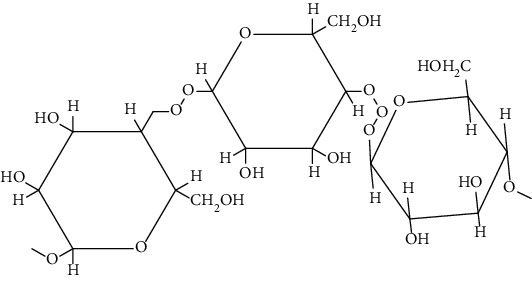	(i) Biocompatibility (ii) Biodegradability (iii) Nontoxicity (iv) Structural similarity with extracellular matrix	(i) High moisture absorption (ii) Dimensional instability
4. *Ganoderma lucidum* It is a medicinal fungus belonging to family Ganodermataceae It has many therapeutic benefits due to which it is known as “mushroom of immortality” It is a pyranoid glucan with beta-glycosidic bond [54].	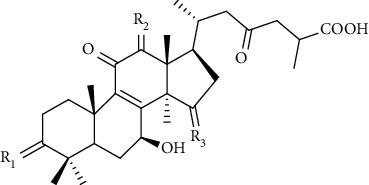	(i) Potent immunomodulatory (ii) Thermal, mechanical and biocompatibility properties	(i) Prone to oxidative damage
5. Konjac gum It is obtained from the tubers of the Amorphophallus konjac plant The structure of konjac gum as residues of mannose and glucose, linked together by *β*-1,4 with a molar ratio of 1.6 : 1.0 [55]	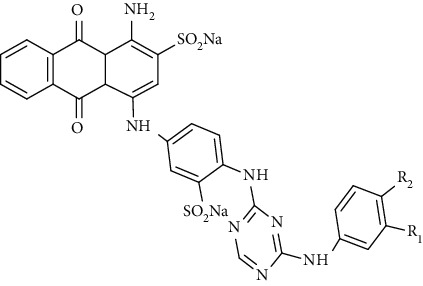	(i) Biocompatible gelling agent (ii) Stimulates immunomodulation and fibroblast proliferation (iii) Excellent water binding and thickening properties	(i) Requires strongly alkaline conditions to form a gel
6. *β*-Glucan It is obtained from mushrooms, yeast products, and whole grains Chemically, it consists of *β*-D-glucose units [56]	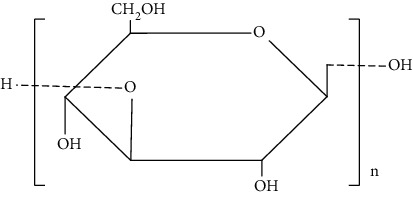	(i) Biocompatible with antihyperglycemic effect (ii) Immunomodulatory (iii) Enhance the migration and proliferation of keratinocytes and fibroblasts toward wound site	(i) Rapid degradation *in vivo*
*Fungus exopolysaccharide*			
7. Pullulan It is obtained from the starch by the fungus *Aureobasidium pullulans* It consists of maltotriose units, i.e., *α*-1,4-;*α*-1,6-glucan [57]	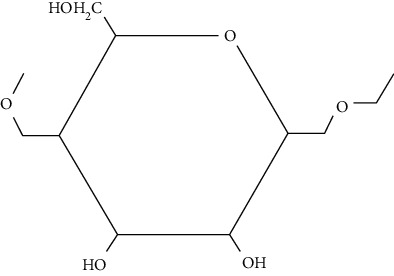	(i) Anticancerous (ii) Tissue rejuvenator (iii) Film forming agent (iv) Plasma expander (v) Grafting (vi) Vaccination	(i) Too expensive and brittle (ii) No antimicrobial property (iii) Inability to provide a surface for supporting cell adhesion
8. Sacchachitin It is a water soluble polysaccharide extracted from *Ganoderma tsugae* and *Ganoderma lucidum* It is composed of 40% chitin and 60% *β*-1,3-glucan [58]	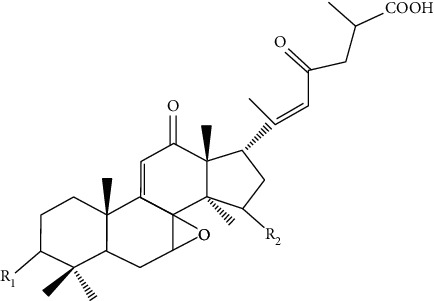	(i) Antitumor activity (ii) Wound healing property	(i) Its purity is still largely limited
9. Scleroglucan It is a branched polysaccharide obtained from the filamentous fungus *Sclerotium rolfsii* It consists of repeating units of *β* (1 → 3)-linked glucose residues with approximately every third residue consist of *β* (1 → 6)-linked D-glucose branch [59]	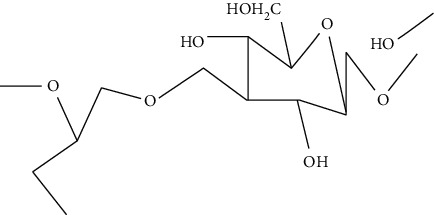	(i) Antitumor, antiviral and antimicrobial activity (ii) Biocompatible (iii) Thermal as well as chemical stability	(i) Tedious preparation (ii) Loss of viscosity during biochemical or chemical reactions
10. Lasiodiplodan It is an exocellular (1→6)-*β*-D glucan produced from Lasiodiplodia theobromae [60]	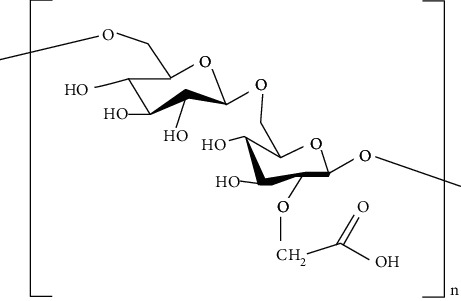	(i) Antioxidant and transaminase activity (ii) Ease of production	(i) Limited control of mechanical properties
*Proteins*			
11. Collagen It is natural polymer obtained from bones, tendons, and ligaments and can be synthesized chemically It consists of three polypeptide chains in its chemical structure [61]	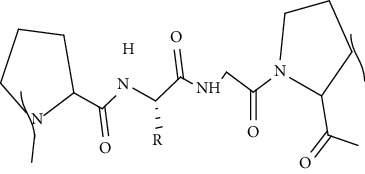	(i) Biodegradability (ii) Low antigenicity (iii) Bio absorbability (iv) High affinity to water (v) Interact with cells through integrin recognition	(i) Insufficient mechanical resistance (ii) Small scale production (iii) Residual salt particles (iv) Slow sublimation of the solvent (v) Lack of control over scaffold topology
12. Fibrin It is derived from fibrinogen It is generally glycosylated with complex type biantennary asparagine-linked glycans [62]	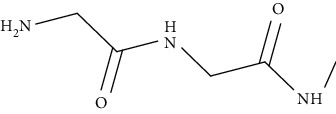	(i) Helps in hemostasis (ii) Elastic and viscous properties (iii) High stability	(i) Rapid degradation
13. Hyaluronic acid It is an anionic, nonsulfated glycosaminoglycan present abundantly in epithelial, connective, and neural tissue It comprises of D-glucuronic acid and N-acetyl- D-glucosamine which is linked by *β*-(1/3) linkages [63]	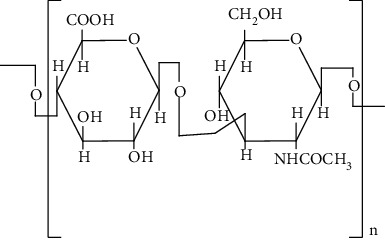	(i) Biocompatible (ii) Low immunogenicity (iii) Wound healing (iv) Analgesic (v) Promote angiogenesis	(i) Poor mechanical properties (iv) Rapid degradation *in vivo*
*Synthetic polymers*			
14. Polylactic acid It is obtained from corn starch or sugar cane It is a biodegredable polyester and made up of lactic acid units [64]	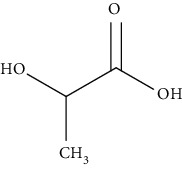	(i) Biodegradable (ii) Form hydrogel under physiological conditions (iii) Allow growth and movement of nutrients	(i) High price (ii) Compostable but not fast enough (iii) Low melting point (iv) Instability
15. Poly-D-L-lactide-glycolide It is synthesized by using copolymerization of glycolic acid and lactic acid [65]	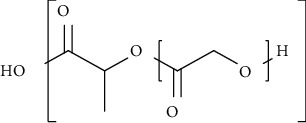	(i) Biodegradability (ii) Biocompatibility (iii) Nontoxic (iv) Helps in local and systemic sustained release of lactate which helps in angiogenesis	(i) Impaired binding affinity after conjugation (ii) High cost
16. Polycaprolactone It is a partial crystalline synthetic polymer formed by the ring-opening polymerization of *ε*-caprolactone [66]	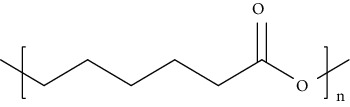	(i) Having low glass transition temperature which assists its biodegradability (ii) Biocompatible (iii) ioresorbable	(i) Weak antimicrobial effect (ii) Complex and expensive production
17. Polyvinyl alcohol It is synthesized by polymerization of vinyl acetate through partial or full hydroxylation [67]	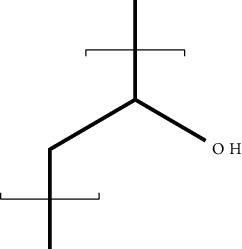	(i) Film former (ii) Adhesive (iii) Emulsifying agent (iv) Biocompatibility (v) Low cytotoxicity	(i) High price (ii) Degradation
18. Polyethylene glycol It is polyether compound derived from petroleum It is made from condensation of ethylene oxide and water [68].	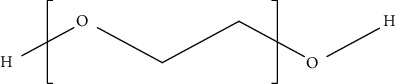	(i) Improves drug solubility (ii) Decreases immunogenicity (iii) Increase drug stability (iv) Increase retention time of conjugates in blood	(i) Low strength and hardness

**Table 4:** Polysaccharide-based scaffolds used for treating DW.

Therapeutic moiety	Method of preparation	Diabetes-inducing agent	Biological model	Key findings	References
*Alginate based scaffolds*					
Cell based	Lyophilization	-	Male Leprdb/db mice	(i) Alginate hydrogel scaffolds composed of macrophages and fibroblast exhibited increase in cell proliferation by 1.7- and 1.8-folds in comparison to hydrogel scaffolds composed of fibroblast alone	[70]
Copper	Irradiation in the presence of photoinitiator	STZ (130 mg/kg)	Male ICR mice	(i) Alginate-PED scaffolds loaded with bioactive glass-based copper showed 1.4- and 1.5-folds increase in wound closure in comparison to alginate-PED-based scaffolds without copper and scaffolds without bioactive glass and copper, respectively	[97]
*Satureja cuneifolia*	Extrusion 3D printer	-	-	(i) Alginate-PEG-based scaffolds loaded with *Satureja cuneifolia* exhibited biphasic pattern with initial burst release followed by controlled release (ii) The developed scaffolds released 96% of the loaded therapeutic moiety in 12 h and provided sustained therapeutic activity for 24 h with improved inflammatory phase	[72, 73]
Silver	Microfluidic spinning and centrifugal reprocessing	-	-	(i) Scaffolds containing calcium alginate inhibited bacterial zone up to 4.9 mm and 5.2 mm against *Escherichia coli* and *Staphylococcus aureus*	[98]
Hydrogen sulphide	Lyophilization	-	Male Wistar rats	(i) Improved epithelization score by 2.2-folds as compared to placebo hydrogel (ii) In addition, promoted angiogenesis and fibroblast migration at the site of injury	[99]
Vitamin D_3_ crosslinked by calcium carbonate/d-glucono-*δ*-lactone	Stirring	-	Male Wistar rats	(i) Vitamin D_3_ hydrogel-based wound dressing exhibited 1.43-folds increase in wound closure in comparison to negative control group (ii) In addition, it promoted neovascularization, angiogenesis, and epithelialization at the site of injury	[100]
*Chitosan-based scaffolds*					
Allicin	Freeze drying	-	Male diabetic rats	(i) Chitosan-based scaffolds using PVA loaded with allicin showed 93.15% wound contraction, 47.11 *μ*m epithelial thickness and 72.31% collagen deposition	[101]
Aloe vera gel	Stirring	-	Male Wistar rats	(i) Improved cell viability and impart antibacterial action at the wound site (ii) Promoted wound healing by showing epithelialization, angiogenesis, fibroplasia, and granulation tissue formation at the site of injury	[102]
bFGF scaffolds	-	-	Female C57BL/6NHsd mice	(i) The developed bFGF scaffolds accelerated DW healing within 10 days as compared to collagen scaffolds alone and control groups	[103]
Cod liver oil	Electrospinning	STZ (60 mg/kg)	Male Wistar albino rats	(i) Wound healing study revealed that chitosan-PLA nanoscaffolds loaded with cod liver oil showed 6-folds decrease in open wound area as compared to groups treated with cod liver oil alone	[104]
Curcumin	Lyophilization	STZ (60 mg/kg)	Male Wistar rats	(i) Chitosan based NPs loaded with curcumin incorporated into collagen-alginate scaffolds exhibited 2.1-folds and 1.5-folds (*p* < 0.01) increase in wound contraction as compared to control and placebo scaffold-treated groups	[95]
dBAM	Lyophilization	STZ (70 mg/kg)	Male C57BL/6 mice	(i) The chitosan-based scaffolds composed of PEGDGE as a crosslinking agent loaded with dBAM (low mass ratio of PEGDGE/dBAM) increased wound healing by 1.3-folds in comparison to free dBAM (ii) The chitosan-based dBAM scaffolds (higher mass ratio of PEGDGE/dBAM) resulted in higher wound healing by 1.2-folds in comparison to chitosan-based scaffolds with lower mass ratio	[105]
Genipin and SDF-1	Freeze-drying	STZ (55 mg/kg)	Male SD rats	(i) Genipin and SDF-1 chitosan-based scaffolds exhibited increase in wound closure within 14 days by 1.09-folds, 1.05-folds, and 1.12-folds in comparison to Genipin-chitosan scaffolds, commercial wound dressing (Comfeel) and gauze control treated groups, respectively (ii) In addition, it promoted angiogenesis, collagen synthesis, and neovascularization at the site of injury	[106]
L-glutamic acid	Lyophilization	STZ (110 mg/kg)	Female Wistar rats	(i) Chitosan hydrogel-based scaffolds loaded with L-glutamic acid increased wound contraction by 2.8- and 1.4-folds in comparison to diabetic control and placebo chitosan hydrogel treated groups	[107]
Pioglitazone	Lyophilization	STZ (60 mg/kg)	Male Wistar albino rats	(i) The results revealed that pioglitazone collagen-chitosan crosslinked scaffolds showed 2.09-folds, 2.42-folds, and 1.05-folds decrease in pioglitazone release in 1 h, 6 h, and 360 h as compared to pioglitazone collagen-chitosan noncrosslinked scaffolds, respectively (ii) This indicated that sustained release of drug from crosslinked scaffold is helpful in combating chronic inflammatory phase of DW	[108]
*Cellulose-based scaffolds*					
Berberine	Lyophilization	STZ (80 mg/kg)	Female SD rats	(i) Cellulose acetate-gelatin-based nanofibrous dressings loaded with berberine exhibited 1.23-folds and 3.01-folds increase in collagen density in comparison to cellulose acetate-gelatin alone and negative control groups. (ii) In addition, exhibited 2.12-folds increase in angiogenesis as (iii) compared to sterile gauze-treated group	[76–78]
*Malva sylvestris*	Electrospinning	STZ (70 mg/kg)	Male Wister rats	(i) CMC-polyurethane-based nanofibrous scaffolds loaded with *Malva sylvestris* exhibited increase in wound closure by 2.9-folds and 1.8-folds as compared to gauze bandage and polyurethane/CMC alone-treated groups	[109]
Pioglitazone	Lyophilization	Alloxan (100 mg/kg)	Male albino Wistar rats	(i) HPMC-chitosan scaffolds composed of pioglitazone decreased the wound area by 1.28-folds and 6-folds wound closure rate in comparison to placebo scaffolds and positive control (diabetic) groups	[110]
Propionyl-L-carnitine	Electrospinning	STZ (55 mg/kg)	Male Wister rats	(i) Propionyl-L-carnitine cellulose-based scaffolds showed 1.1-folds increase in wound contraction within 14 days in comparison to placebo scaffolds	[111]
*Konjac glucomannan-based scaffolds*					
*Avena sativa*	Lyophilization	STZ (50 mg/kg)	Male Wister rats	(i) Konjac glucomannan-keratin hydrogel scaffold loaded with *Avena sativa* increased the collagen content by 1.4-folds in comparison to placebo scaffolds	[79, 80]
*β-Glucan-based scaffolds*					
*β*-Glucan	-	-	Male ICR mice	(i) *In vivo* wound healing study showed that *β*-glucan-loaded nanofibers exhibited 2.9-folds increase in wound closure as compared to diabetic control group (ii) Accelerated DW healing by promoting reepithelialization, collagen deposition, and angiogenesis at the site of injury	[81–84]
*Psyllium-based scaffolds*					
Morin + psyllium+ keratin	Freeze drying	STZ (50 mg/kg)	Male Wistar albino rats	(i) Morin-psyllium-keratin-based hydrogel scaffolds increased wound contraction by 1.42-folds and 1.66-folds in comparison to combination of psyllium and keratin and diabetic control groups	[112]

Abbreviations: bFGF: basic fibroblast growth factor; CA: calcium alginate; CMC: carboxy methyl cellulose; dBAM: decellularized bovine amniotic membrane; DW: diabetic wound; HPMC: hydroxy propyl methyl cellulose; ICR: institute of cancer research; PLGA: poly-D-L-lactide-glycolide; PLA: polylactic acid; PED: polyethylene glycol diacrylate; STZ: streptozocin; SDF-1: stromal-derived factor-1.

**Table 5:** Applications of protein-based scaffolds for DW.

S.N.	Therapeutic moiety	Method of preparation	Diabetes-inducing agent (dose)	Animal model	Key findings	References
*Collagen-based scaffolds*						
1.	Adipose-derived SVFs	Freeze drying	STZ (125 mg/kg)	Female domestic pigs	(i) Scaffolds containing adipose-derived SVFs showed 2.04-folds and 1.79-folds increase in blood vessel density in comparison to diabetic control and SVFs alone-treated groups	[135]
2.	Bcl-2-modified ADSCs	Extraction and purification	STZ (165 mg/kg)	Female db/db mice	(i) Scaffolds loaded with Bcl-2-modified ADSCs exhibited 1.6-folds and 2-folds increase in wound closure as compared to placebo scaffolds and PBS alone-treated groups, respectively	[121]
3.	bFGF	Freeze drying	-	Female BKS.Cg– + Leprdb/+ Leprdb/Jcl mice	(i) Scaffolds loaded with bFGF (14 *μ*g/cm^2^) showed 7.8-folds, 3.5-folds, 5-folds, and 5.5-folds decrease in wound area within 2 weeks in comparison to collagen scaffolds loaded with normal saline solution, collagen scaffolds loaded with bFGF (7 *μ*g/cm^2^), collagen scaffolds loaded with bFGF (28 *μ*g/cm^2^), and collagen scaffolds loaded with bFGF (50 *μ*g/cm^2^)	[136]
4.	BM-MSCs	Lyophilization	STZ (65 mg/kg)	Male Wistar rats	(i) Scaffolds loaded with BM-MSCs showed decrease in wound area by 1.2-folds, 1.8-folds, and 2-folds under hypoxic condition in comparison to collagen-based scaffolds loaded with BM-MSCs under norxomia, placebo scaffolds, and diabetic control groups (ii) This developed scaffold increased angiogenesis and anti-inflammatory action at the wound site	[137]
5.	Collagen	Electrospinning	STZ (65 mg/kg)	Male SD rats	(i) Crossed collagen-polycaprolactone-based scaffolds showed 1.1-folds, 1.2-folds, and 1.7-folds increase in wound contraction as compared to aligned collagen-polycaprolactone-based scaffolds, random collagen-polycaprolactone-based scaffolds, and diabetic control treat groups, respectively (ii) The developed collagen-loaded crossed scaffolds induced reepithelialization, angiogenesis, migration of keratinocytes as well as fibroblasts at the site of injury that promoted DW healing	[138]
6.	Glucophage	Electrospinning	STZ (70 mg/kg)	Male SD rats	(i) Collagen-PLGA scaffolds loaded with nanofibrous glucophage exhibited 6.7-folds and 1.9-folds decrease in wound closure as compared to collagen/PLGA membranes and diabetic control group, respectively	[139]
7.	Induced pluripotent stem cells	Homogenization	STZ (50 mg/kg)	Male athymic nude mice	(i) Scaffolds loaded with induced pluripotent stem cells exhibited 1.44-folds and 1.28-folds increase in epidermal thickness and muscle thickness in comparison to adipose-derived stem cells and mesenchymal stem cell-treated groups	[140]
8.	Induced pluripotent stem cell	Freeze drying	-	Human fibroblasts	(i) Induced pluripotent stem-based scaffolds seeded on DFU fibroblast cells showed higher content of vascular endothelial GFs and glycosaminoglycan that helped in production of extracellular matrix	[141]
9.	MSCs	Homogenization	STZ (50 mg/kg)	Male C57BL/6 mice	(i) Topical application of MSCs-based scaffolds accelerated wound closure by 1.3-folds as compared to MSCs alone-treated groups due to increased cell proliferation, angiogenesis, and collagen deposition at the wound site	[142]
10.	Mesenchymal stromal cells	Lyophilization	Alloxan (150 mg/kg)	Male New Zealand white rabbits	(i) The scaffolds loaded with allogenic mesenchymal stromal cells exhibited 1.1-folds, 1,2-folds, 1.2-folds, and 1.1-folds increase in volume of inflammatory cells, surface density of blood vessels, surface area of blood vessels, and vessel diameter as compared to collagen alone treated, groups respectively	[143]
11.	N-acetylcysteine	Lyophilization	-	Male SD rats	(i) Scaffolds loaded with polyamide and N-acetylcysteine exhibited 1.5-folds and 1.4-folds increase in drug release in a sustained manner as compared to scaffolds composed of collagen and N-acetylcysteine (ii) Collagen-polyamide-based scaffolds coloaded with N-acetylcysteine showed 1.5-folds increase in wound closure as compared to polyamide treated groups	[144]
12.	Osteopontin	Lyophilization	Alloxan (150 mg/kg)	Male New Zealand white rabbits	(i) Circulating angiogenic cells-osteopontin collagen-based scaffolds exhibited 1.2- and 1.3-folds increase in wound closure as compared to collagen scaffolds loaded with circulating angiogenic cells and collagen alone-treated groups	[145]
13.	PHB and gelatin	Electrospinning	STZ (60 mg/kg)	Male Wistar rats	(i) PHB- and gelatin-based scaffolds showed 2-folds, 5-folds, and 5.5-folds decrease in wound area within 14 days as compared to gelatin nanofibers, PHH microfibers, and diabetic control group, respectively (ii) In addition, the developed scaffolds increased collagen synthesis, cell attachment, and proliferation at the site of injury	[146]
14.	Quercetin	Fat digestion	STZ (80 mg/kg)	Male ICR mice	(i) Collagen scaffolds loaded with PEGylated graphene oxide and quercetin exhibited 2-folds, 1.6-folds, and 1.3-folds decrease in drug release at pH 7.4 within 15 h, 25 h, and100 h as compared to ADM scaffolds composed of graphene oxide and quercetin (ii) The scaffolds composed of PEGylated graphene oxide and quercetin showed 1.28-folds increase in wound closure as compared to ADM treated group	[147]
15.	Resorcinol	Freeze drying	STZ (55 mg/kg)	Male albino rats	(i) Resorcinol-loaded collagen-based scaffolds exhibited increase in wound closure by 1.25-folds as compared to collagen alone-treated group due to reepithelization, angiogenesis, and collagen deposition at the wound site	[148]
16.	siMMP-9	Freeze drying	-	-	(i) Glycosaminoglycan collagen scaffolds loaded with siMMP-9 (80 nmol/L) treated groups showed 3.3-folds, 3-folds, 1.5-folds, and 1.3-folds decrease in relative MMP-9 mRNA expression in comparison to diabetic control, nontargeted siRNA, glycosaminoglycan collagen scaffolds composed of siMMP-9 (20 nmol/L), and glycosaminoglycan collagen scaffolds composed of siMMP-9 (40 nmol/L) treated groups	[149]
17.	VEGF	Lyophilization	STZ (50 mg/kg)	Male SD rats	(i) Collagen scaffolds loaded with VEGF showed decrease in wound closure within 21 days by 1.06-folds, 1.14-folds, and 1.18-folds in comparison to VEGF alone, PBS, and diabetic control-treated groups	[150]
*Fibrin-based scaffolds*						
18.	eNOS	Homogenization	Alloxan (150 mg/kg)	New Zealand white rabbits	(i) Fibrin scaffolds loaded with eNOS exhibited 1.26-folds and 1.46-folds increase in epithelialization rate as compared to eNOS alone and fibrin alone-treated groups	[151]
*Hyaluronic acid-based scaffolds*						
19.	Chlorhexidine	-	-	Male albino rats	(i) Hyaluronic acid scaffolds loaded with chlorhexidine exhibited increase in wound closure by 1.2-folds and 1.09-folds as compared to gauze and SEESKIN® (clinically used scaffold in market)-treated groups (ii) The total protein content of hyaluronic acid scaffolds loaded with chlorhexidine treated group was found to be 1.2-folds higher in 21 days than that of SEESKIN®-treated group	[152]

Abbreviations: ADSCs: adipose-derived stem cells; Bcl-2: B-cell lymphoma-2; BM-MSCs: bone marrow-derived mesenchymal stem cells; bFGF: basic fibroblast growth factor; eNOS: endothelial nitrous oxide synthase; ICR: institute cancer research; MMP-9: matrix mettalo proteinase-9; PLGA: poly-D-L-lactide-glycolide; PHB: poly-3-hydroxybutyrate; SVFs: stromal vascular fraction; VEGF: vasoendothelial growth factor.

**Table 6:** Applications of synthetic polymer-based scaffolds for DW.

S.N.	Therapeutic moiety	Method of preparation	Diabetes-inducing agent	Animal model	Key findings	References
*Polylactic acid-based scaffolds*						
1.	Cod liver oil	Microwave irradiation	STZ (60 mg/kg)	Male Netherlands rats	(i) Scaffolds loaded with cod liver oil showed 6-folds decrease in open wound area as compared to cod liver alone-treated group	[181]
2.	EGF	Electrospinning	-	Female Kunming mice	(i) Scaffolds loaded with EGF exhibited 1.25-folds and 1.75-folds increase in wound closure as compared to PLA-gelatin alone and diabetic control groups due to increased reepithelization and angiogenesis at the site of injury	[182]
3.	Monocyte chemoattractant protein-1	Electrospinning	STZ (100 mg/kg)	Female C57BL/6 mice	(i) DES containing monocyte chemo attractant protein-1 showed 1.4-folds faster wound healing as compared to diabetic control group due to enhanced reepithelization at the wound site	[183]
4.	PLA-CS	Electrospinning	STZ (60 mg/kg)	Male diabetic rats	(i) The prepared scaffolds exhibited 2.25-folds and 1.57-folds increase in wound closure as compared to diabetic control and placebo scaffolds alone-treated groups, respectively	[184]
*Poly-D-L-lactide-glycolide-based scaffolds*						
5.	Asiatic acid	Electrospinning	STZ (40 mg/kg)	Male C57BL/6J mice	(i) Scaffolds containing asiatic acid-induced angiogenesis, ECM remodelling, and reepithelization at the wound site that helped in DW healing (ii) In addition, it exhibited 1.8-folds increase in wound closure as compared to PLLA-treated groups	[185]
6.	Curcumin	Electrospinning	STZ (80 mg/kg)	Male SD rats	(i) Scaffolds loaded with curcumin exhibited 6.8-folds increase in curcumin release in a sustained manner as compared to raw curcumin in 500 h that helped in retaining drug at the wound site for a prolonged period of time (ii) In addition, it exhibited 1.47-folds, 1.97-folds, and 2.6-folds increase in wound closure as compared to combination of PLGA with curcumin, PLGA alone, and gauze treated groups, respectively	[186]
6.	Glucophage	Electrospinning	STZ (70 mg/kg)	Male SD rats	(i) Scaffold loaded with glucophage exhibited 6-folds and 2-folds decrease in wound area as compared to collagen/PLGA nanofibrous membrane and conventional gauze sponge-treated groups	[187]
7.	HB-EGF	Electrospinning	STZ (70 mg/kg)	Male SD rats	(i) 26 SCS PLGA-based scaffolds loaded with HB-EGF showed 1.2-folds increase in HB-EGF in a controlled manner as compared to PLGA alone scaffolds in 10 days that helped in combating inflammatory phase of DW (ii) 26SCS PLGA-based scaffold-treated groups exhibited 1.18-folds increase in wound closure as compared to diabetic control group	[188]
8.	Insulin	Coaxial electrospinning	STZ (70 mg/kg)	Male SD rats	(i) Scaffolds loaded with insulin exhibited about 8.5 mU/mL of insulin release in 28 days in a sustained manner that can help in combating chronic inflammatory phase of DW (ii) Scaffolds loaded with insulin exhibited 5.4-folds and 2.9-folds decrease in TGF-*β* levels as compared to insulin glargine and phosphate buffer saline-treated groups	[189]
9.	Liraglutide	Electrospinning	STZ (65 mg/kg)	Male SD rats	(i) Scaffolds loaded with liraglutide exhibited 1.17-folds and 1.33-folds increase in wound closure as compared to placebo scaffolds and diabetic control groups, respectively	[190]
10.	Neurotensin	Electrospinning	Mutation in leptin receptor	Female BKS.Cg-Dock7m^+/+^Lep rdb/JNju mice	(i) Scaffolds loaded with neurotensin exhibited 1.38-folds increase in wound closure as compared to diabetic control group	[191]
11.	Recombinant human platelet-derived growth factor	Electrospinning	STZ (70 mg/kg)	Male SD rats	(i) Scaffolds loaded with recombinant human-derived platelet GF exhibited 2-folds and 1.9-folds decrease in wound area as compared to collagen alone and PLGA alone-treated groups	[192]
*Polycaprolactone- (PCL-) based scaffolds*						
12.	Bixin	Electrospinning	STZ (40 mg/kg)	Male C57BL/6 mice	(i) Nanofibrous scaffolds loaded with bixin exhibited 1.2-folds increase in wound closure as compared to PCL alone-treated group (ii) In addition, it accelerated DW healing by promoting reepithelialization, angiogenesis, and collagen deposition at the site of injury	[193]
13.	Collagen-PCL and bioactive glass NP-based scaffolds	Electrospinning	STZ (65 mg/kg)	Male SD rats	(i) Scaffolds containing bioactive glass NPs exhibited 1.05 and 1.1-folds increase in wound closure as compared to placebo scaffolds and diabetic control (untreated) groups (ii) In addition, it exhibited complete wound closure within 21 days due to enhanced collagen matrix remodelling and reepithelialization at the wound site	[194]
14.	Curcumin	Electrospinning	STZ (60 mg/kg)	Male SD rats	(i) Scaffolds loaded with curcumin exhibited 65 % of curcumin release in a sustained manner for 20 days that prolonged the retention of drug at the wound site (ii) In addition, it showed 3.1-folds decrease in wound area as compared to diabetic control group (iii) Furthermore, it accelerated DW healing within 15 days by promoting collagen deposition, angiogenesis, antibacterial action, reepithelization, as well as regeneration of sweat glands and hair follicles at the wound site	[195]
15.	Curcumin	Electrospinning	STZ (70 mg/kg)	Male SD rats	(i) Scaffolds loaded with curcumin showed 2.6-folds and 2.8-folds increase in wound closure as compared to placebo scaffolds (positive control) and 5% treated with sodium lauryl sulfate solution (negative control) groups (ii) In addition, it accelerated DW healing by promoting anti-inflammatory and antioxidant action at the wound site	[196]
*PVA-based scaffolds*						
16.	Cephradine	Electrospinning	-	Male NcZ10 mice	(i) Scaffolds loaded with cephradine exhibited 1.08-folds increase in drug release in a sustained manner within 24 h in comparison to free cephradine that helped in DW healing by increasing retention time of drug at the wound site (ii) In addition, it showed 2-folds decrease in wound area as compared to cephradine alone-treated groups	[197]
17.	Desferrioxamine	Electrospinning	STZ (70 mg/kg)	Male SD rats	(i) Scaffolds loaded with desferrioxamine exhibited 92.7% of desferrioxamine release within 72 h in a sustained manner that can prolong drug retention at the wound site (ii) In addition, it exhibited 20-folds faster wound closure as compared to placebo scaffolds (iii) Furthermore, it enhanced cell proliferation, cell migration, and neovascularization at the site of injury that accelerated the wound healing	[198]

Abbreviations: BM-MSCs CCSS: bone marrow-derived MSC-based collagen and chitosan sponge scaffolds; Cur: curcumin; DES: drug eluting scaffolds; EGF: epidermal growth factor; GT: gum tragacanth; PCL: poly (*ε*-caprolactone); SC: sacchachitin; 26-SCS: 2-*N*, 6-*O* sulfated chitosan.

**Table 7:** Different techniques of scaffold fabrication.

Fabrication technique	Advantages	Disadvantages	References
*Conventional technique*			
Thermal-induced phase separation	(i) Used for the fabrication of thermoplastic crystalline polymeric scaffold (ii) Bioactive molecules can be integrated by utilizing low temperature (iii) Provide porosity to the fibers more than 98%	(i) Fabricate only thermoplastic polymeric scaffolds	[199, 200]
Electrospinning	(i) Develop nanofibrous scaffolds (ii) Improve tensile strength of the scaffolds	(i) Solvent used in the fabrication can be toxic (ii) Require lot of process variables	
Gas foaming	(i) Provide porosity to the fibers upto 85%	(i) Product obtained might have a closed pore structure or a solid polymeric skin	
Solvent casting and practical leaching	(i) Cost effective (ii) High porosity upto 50-90%	(i) Time consuming (ii) The widespread use of the toxic solvents	
Freeze-drying	(i) Utilized in variety of purposes (ii) Manageable pore size	(i) High energy consumption (ii) Time consuming (iii) Use of cytotoxic solvents in the fabrication	
*RP technique*			
Fused deposition modeling (FDM)	(i) Useful in the scaffold designing (ii) Low-temperature deposition	(i) Has limitations in its application to biodegradable polymers	[199, 200]
Stereolithography (SLA)	(i) High resolution (ii) Uniformity in the pore size interconnectivity	(i) Limitations in the process of photopolymerization (ii) Requiring massive amounts of monomers and postpolymerization treatment to improve monomer conversion	
Selective laser sintering (SLS)	(i) Using ultrahigh-molecular-weight polyethylene (ii) Fabricate scaffold with preferred properties (iii) Provide excellent microstructures	(i) High operating temperature (ii) Time consuming	
Solvent-based extrusion free forming (SEF)	(i) Utilize in the fabrication of ceramic and metal-based composites (ii) Provide precise control of scaffold structure at the micron level	(i) Temperature extrusion	
Bioprinting	(i) Cost effective (ii) High accuracy (iii) Greater shape complexity (iv) Higher speed	(i) Depends on existence of cells	

**Table 8:** Electroconductive scaffolds for DW.

Polymer	Delivery system	Outcomes	Pitfalls	References
*Nerve tissue*				
Aniline/polylactide/Pu	Nanofibrous composite scaffolds	(i) Myoblast proliferation and differentiation	(i) Poor biodegradability	[206–208]
Alginate-aniline tetramer/agarose	Hydrogel-based scaffold	(i) Cell proliferation (ii) Ionic conductivity (iii) Enhanced cell viability	(i) Issues with large-scale reproducibility	
Polyethylene terephthalate/graphene	Sheet-based scaffolds	(i) Increased interaction at the site of neuroblastoma cells	(i) Only applicable for *in vitro* studies	
Graphene	Foam-based scaffolds	(i) Promoted neural stem cell growth (ii) Upregulation of Ki67 expression	(i) Scalability issues	
Carbon/polylactic acid	Nanofibrous scaffolds	(i) Enhanced osteoblast alkaline phosphatase activity (ii) Promoted collagen type I synthesis of osteoblasts	(i) Only applicable for *in vitro* studies	
Carbon	Nanofibrous scaffolds	(i) Upregulation of signaling-related kinases (ii) Proliferating nuclear antigens	(i) The results may only be applicable for one specific cell type with potential outcomes for nerve cells	
Carbon	Nanofibrous scaffolds	(i) Augmented neurite outgrowth	-	
PEGylated-reduced graphene Oxide	Sheet-based scaffolds	(i) Desirable cell microenvironment	(i) Scalability issues	
Polyhedral oligomeric silsesquioxanes/polyurethane/polycaprolactone	Carbon nanotube-based scaffolds	(i) Promote neuronal regeneration	(i) Cytotoxicity	
*Heart*				
Polyaniline-gelatin	Nanofibrous scaffolds	(i) Enhance cardiac differentiation	-	[206–208]
Polyethersulfone and polyaniline	Nanofibrous scaffolds	(i) Enhance cardiomyocyte differentiation, proliferation, and adhesion	(i) Poor biodegradibility and blending	
Polyaniline-polylactic acid	Nanofibrous scaffolds	(i) Induce cardiac differentiation, maturation, and spontaneous beating of seeded cells (ii) Promote cell-cell interactions	(i) Poor biodegradibility	
Polylactic glycolic acid	Carbon nanofibrous scaffolds	(i) Improved cardiomyocyte density	(i) Only applicable for *in vitro* studies	
Polydimethylsiloxane	Carbon nanotube-based scaffolds	(i) Enhanced viability of cell culture (ii) Improved maturation of ventricular myocytes in neonatal rats	(i) Toxicity challenge with carbon nanotubes	
*Bone/skin*				
Polyaniline/polycaprolactone	Nanofibrous scaffolds	(i) Improved biocompatibility and conductivity that helps in tissue rejuvenation	(i) The proposed follow-up has not been successfully done	[206–208]
Graphene	Hydrogel-based scaffolds	(i) Enhanced cell proliferation, adhesion, and differentiation	-	
Chitosan	Nanofibrous scaffolds	(i) Supported cell growth and attachment	(i) Potential batch-to-batch variation	
Chitosan	Hydrogel-based scaffolds	(i) Enhanced inflammatory response	-	

**Table 9:** Outcomes of some clinical trials conducted on marketed scaffolds for wound healing.

S.no.	Scaffolds type	Marketed formulation	Manufacturer	Source	No. of patients	Clinical outcomes	References
1.	Acellular	PriMatrix	TEI Biosciences	Bovine	55	(i) The marketed formulation showed complete wound closure in 46 patients within 12 weeks by showing collagen deposition and epithelialization at the site of injury	[210]
2.	Acellular	Biobrane	Smith & Nephew UK Limited	Porcine	34	(i) It exhibited 1.8 days faster epithelization rate as compared to suprathel dressing	[211]
3.	Acellular	AlloDerm	Biohorizon	Donated human skin	77	(i) The topical application of AlloDerm scaffolds provide proper coverage to the wound and promoted collagen deposition and elastin at the site of injury that facilitates wound healing	[212]
4.	Acellular	Graftjacket	Wright medical group	Cadaver human skin	28	(i) Graftjacket showed 3-folds increase in wound healing rate as compared to debriment therapy	[213]
5.	Acellular	MatriDerm	MedSkin Solutions	Bovine dermis	60	(i) MatriDerm showed 1.18-folds increase in reepithelization as compared to skin graft therapy	[214]
6.	Acellular	Permacol	Medtronic	Porcine dermis	343	(i) The formulation induced angiogenesis, epithelialization, and collagen deposition at the site of injury that helped in wound healing	[215]
7.	Collagen	Apigraft	Organogenesis	Bovine	30	(i) It exhibited 1.28-folds increase in wound closure as compared to diabetic control group	[216]
8.	Collagen	Terudermis	Olympus Termo Biomaterials Corp.	Bovine/synthetic	30	(i) The developed scaffolds promoted neovascularization and perforation closure that accelerated wound healing	[217]
9.	Collagen	Orcel	Ortec International	Bovine	82	(i) It exhibited 0.8-fold faster wound closure than Biobrane-L-treated groups	[218]
10.	Collagen	Promogran Prisma ® Matrix	Systagenix Wound Management	Bovine	25	(i) Topical application of prepared scaffolds showed 1.83-folds increase in wound closure within 4 weeks as compared to diabetic control group	[219]
11.	Collagen	EZ Derm	Molnlycke Health Care	Porcine	157	(i) EZ Derm promoted epithelization, antibacterial action, and moist environment at the site of injury and resulted in wound closure	[220]
12.	Collagen crosslinked glycosaminoglycan polysiloxane	Integra dermal regeneration template	Integra Life Sciences	Bovine/synthetic	307	(i) It showed 1.57-folds increase in wound closure as compared to offloading therapy	[221]
13.	Decellularized	OASIS wound matrix	Cook Biotech, Inc.	Porcine small-intestine submucosa	120	(i) The developed scaffolds showed 1.6-folds increase in wound closure as compared to standard of care	[222]
14.	HA	Hyalograft 3D	Fidia Advanced Biopolymers SRL	Allogenic	180	(i) HA-based scaffolds exhibited 1.16-folds increase in ulcer healing within 20 weeks as compared to nonadherent paraffin gauze	[223]
15.	HA	Hyalomatrix PA	Medline Industries, Inc.	Allogenic/synthetic	262	This marketed product promoted wound healing within 16 days by showing reepithelization at the site of injury	[224]
16.	NAG	Talymed	Marine Polymer Technologies, Inc.	Microalgae	82	Talymed exhibited 1.92-folds increase in wound closure within 3 weeks as compared standard of care-treated groups	[225]
17.	PLGA and collagen	Cytoplast	Osteogenics	Bovine	1	The developed market scaffolds showed MSCs and osteoprogenitor cell regeneration that promoted wound closure	[226]
18.	PGA/PLA	Dermagraft	Organogenesis Inc.	Synthetic	130	Dermagraft-based treatment exhibited 1.63-folds increase in wound closure within 12 weeks as compared to conventional wound care therapy	[227, 228]

HA: hyaluronic acid; NAG: N-acetyl glucosamine; PLGA: poly-D-L-lactide-glycolide; PGA/PLA: polyglycolic/polylactic acid.

**Table 10:** Patents on scaffolds for wound healing.

S. no.	Therapeutic moiety	Claims	Patent number	Outcome	References
1.	Alimentary protein	(i) Electroprocessed-based composition based on plant-derived fiber and synthetic polymer to treat wound	US20170319743A1	(i) The prepared scaffolds promoted cell migration and proliferation at the site of injury that helped in wound healing	[229]
2.	Amniotic membrane	(i) The composition based on amniotic membrane rich in cytokines and extracellular matrix protein to treat DW	EP2897625B1	(i) Amniotic membrane-based scaffolds increased collagen deposition, angiogenesis, and cell migration at the site of injury	[230]
3.	DEM	(i) The composition comprised DEM and lipid-derived from fish skin for tissue rejuvenation in treating DW	US8613957B2	(i) DEM accelerated wound healing by promoting cell migration, cell differentiation, and proliferation and facilitated replacement of tissues	[231]
4.	ECM	(i) The composition containing ruminant forestomach isolated extracellular matrix to treat DW	US8758781B2	(i) DFM showed 1.6-folds increase in angiogenesis as compared to subintestinal mucosa matrix	[232]
5.	Magnesium-PLGA	(i) The composition based on magnesium and polymer composite for tissue rejuvenation in treating DW	US20170014548A1	(i) Magnesium-PLGA-based scaffolds showed 1.6-folds increase in cell proliferation within 6 days as compared to PLGA alone	[233]
6.	MSCs	(i) The MSC-based scaffolds to treat wound	WO2008060374A3	----------------------------	[234]
7.	Silk fibroin	(i) Composition based on porous and impermeable layers and methods relating thereto to treat DW	US20210178017A1	(i) Silk fibroin-based scaffolds promoted angiogenesis at the site of injury that helped in wound healing	[235]
8.	Sodium alginate and gelatin	(i) Method of formation of scaffolds based on 3D-bioprinting technique and its composition containing sodium alginate, gelatin, and calcium chloride to treat wounds	CN113181419A	(i) Sodium alginate-gelatin-based scaffolds increased the rate of epithelization within 14 days and promoted DW healing	[236]
9.	Stem cells	(i) The composition of scaffolds containing collagen, alginate, polyglycolic acid, and polyglactin to treat wound	JP2007275613A	(i) Stem cell-loaded scaffolds healed wound within 8 weeks by promoting fibers and epithelial cell formation at the site of injury	[237]
10.	PCL	(i) The composition of scaffolds comprising polyglycolic acid, poly (lactide-co-caprolactone), polylactic acid, polycaprolactone, and copolymers to treat wound	US20200179437A1	(i) PCL-based scaffolds showed 70% decrease in planimetric area within 42 days due to angiogenesis and collagen deposition at the site of injury	[238]

DFM: decellularized forestomach membrane; DEM: decellularized extracellular matrix; MSC: mesenchymal stem cell; PCL: polycaprolactone; PLGA: poly-D-L-lactide-glycolide.
